# MitoRS, a method for high throughput, sensitive, and accurate detection of mitochondrial DNA heteroplasmy

**DOI:** 10.1186/s12864-017-3695-5

**Published:** 2017-04-26

**Authors:** Julien Marquis, Gregory Lefebvre, Yiannis A. I. Kourmpetis, Mohamed Kassam, Frédéric Ronga, Umberto De Marchi, Andreas Wiederkehr, Patrick Descombes

**Affiliations:** 1 Functional Genomics, Nestlé Institute of Health Sciences, 1015 Lausanne, Switzerland; 2 Digital Nutrition and Health, Nestlé Institute of Health Sciences, 1015 Lausanne, Switzerland; 3 Mitochondrial Functions, Nestlé Institute of Health Sciences, 1015 Lausanne, Switzerland

**Keywords:** Next generation sequencing, Mitochondria, Mitochondrial DNA, Heteroplasmy, Rolling circle amplification, Somatic mutation, Low frequency variant, MitoRS

## Abstract

**Background:**

Mitochondrial dysfunction is linked to numerous pathological states, in particular related to metabolism, brain health and ageing. Nuclear encoded gene polymorphisms implicated in mitochondrial functions can be analyzed in the context of classical genome wide association studies. By contrast, mitochondrial DNA (mtDNA) variants are more challenging to identify and analyze for several reasons. First, contrary to the diploid nuclear genome, each cell carries several hundred copies of the circular mitochondrial genome. Mutations can therefore be present in only a subset of the mtDNA molecules, resulting in a heterogeneous pool of mtDNA, a situation referred to as heteroplasmy. Consequently, detection and quantification of variants requires extremely accurate tools, especially when this proportion is small. Additionally, the mitochondrial genome has pseudogenized into numerous copies within the nuclear genome over the course of evolution. These nuclear pseudogenes, named NUMTs, must be distinguished from genuine mtDNA sequences and excluded from the analysis.

**Results:**

Here we describe a novel method, named MitoRS, in which the entire mitochondrial genome is amplified in a single reaction using rolling circle amplification. This approach is easier to setup and of higher throughput when compared to classical PCR amplification. Sequencing libraries are generated at high throughput exploiting a tagmentation-based method. Fine-tuned parameters are finally applied in the analysis to allow detection of variants even of low frequency heteroplasmy. The method was thoroughly benchmarked in a set of experiments designed to demonstrate its robustness, accuracy and sensitivity. The MitoRS method requires 5 ng total DNA as starting material. More than 96 samples can be processed in less than a day of laboratory work and sequenced in a single lane of an Illumina HiSeq flow cell. The lower limit for accurate quantification of single nucleotide variants has been measured at 1% frequency.

**Conclusions:**

The MitoRS method enables the robust, accurate, and sensitive analysis of a large number of samples. Because it is cost effective and simple to setup, we anticipate this method will promote the analysis of mtDNA variants in large cohorts, and may help assessing the impact of mtDNA heteroplasmy on metabolic health, brain function, cancer progression, or ageing.

**Electronic supplementary material:**

The online version of this article (doi:10.1186/s12864-017-3695-5) contains supplementary material, which is available to authorized users.

## Background

Mitochondria carry a small circular double-stranded genome of 16’569 bp in human which encodes the mitochondrial 16S and 12S ribosomal RNA, 22 mitochondrial tRNA molecules and 13 proteins of the respiratory chain. Therefore only a minute fraction of a total of about 1’500 mitochondrial proteins is encoded by the mitochondrial DNA (mtDNA) whereas all other proteins are nuclear DNA encoded and imported into mitochondria [1, 2]. Mitochondrial DNA also encodes for some short peptides which roles and mechanisms of action are not fully understood [3]. Common non-pathogenic mtDNA variations can be classified in so-called haplogroups, defining specific populations that can be linked to their maternal lineage [4]. The relatively high mutation rate of mitochondrial DNA makes haplogroup determination and classification an important tool for paleoanthropology and population genetics. Because of the large degree of sequence variability, maternal mode of inheritance and biological particularities (such as high copy number per cell and presence in enucleated cells), mtDNA analysis is also widely used in forensic science [5].

The mitochondrial genome is present in multiple copies ranging from a few hundred to several thousand copies per cell. Consequently, a mutation occurring in the mitochondrial DNA results in a sequence heterogeneity within the mtDNA pool. Several mtDNA populations can therefore co-exist in a cell, a phenomenon called heteroplasmy. The extent of heteroplasmy can be highly variable even within an organism. The concept of heteroplasmy is known from a long time but many recent findings resulting from sensitive variant detection approaches highlighted how far this is a common situation [6–12]. Note that without single cell resolution, it is not possible to distinguish heteroplasmy at individual cell level from an apparent heteroplasmy which would be the result of the average of several cells with a distinct mtDNA content. Some heteroplasmic mtDNA mutations are actually somatic, i.e. they are not inherited but occur over the life course. Accordingly, many studies report an increasing number and frequency of heteroplasmic mtDNA variants with age [10, 12, 13]. This is also a factor contributing to the tissue heterogeneity for heteroplasmy [10].

Mitochondrial DNA mutations can impair oxidative phosphorylation and therefore give rise to primary mtDNA-related diseases. Today, several hundred mtDNA point mutations affecting every mtDNA encoded gene have been identified [2]. About 1 in 5’000 individuals develop an mtDNA-related disease, while the frequency of carriers of mtDNA mutations may be much higher. For instance, 1 in 200 healthy humans was found to carry one of the 10 most abundant pathogenic mtDNA mutations [14]. Clinical symptoms consecutive to these mutations are usually observed in muscle, heart, endocrine or brain, which are all tissues strongly depending on mitochondria for energy production. Individuals with mitochondrial diseases are usually heteroplasmic carrying a mixture of wild-type and mutated mitochondrial genomes [11, 15, 16]. Clinical manifestation therefore does not only depend on the specific mutation and the affected gene but also on the ratio of mutated to wild-type mtDNA. Exceeding a ratio threshold leads to energy stress in the vulnerable tissues and consequently to a variety of disease symptoms (for example, see an elegant molecular analysis by Picard et al. [17]). Recent studies highlighted that this threshold can be of first importance for nuclear reprogramming since asymptomatic low frequency heteroplasmy variants present in the donor cells can turn into deleterious high heteroplasmy variants into some of the derived induced pluripotent stem cell clones (iPSC) [12, 18]. Accurate and sensitive methods for the analysis of mtDNA are therefore necessary to enable the detection and quantification of low frequency heteroplasmy variants.

Mitochondrial DNA analysis is further complicated by the presence of nuclear DNA (nucDNA) regions homologous to mtDNA. These regions, called NUMTs (for Nuclear MiTochondrial DNAs), are the result of an extensive mtDNA pseudogenization in the nuclear genome during evolution. They can be found as blocks of several kilobases, highly homologous to the genuine mtDNA sequence, and spread as multiple copies throughout the genome. Some NUMTs seems to be universal, whereas others may be specific to some subpopulations [19].

Human and mouse mtDNA have been fully sequenced in the early 80s [20, 21]. Mitochondrial DNA sequencing was historically carried out using Sanger sequencing of PCR products. Several protocols have been described for human mtDNA, the most classical ones involving ~ 30 distinct PCR reactions to cover the ~ 16.5 kb genome. PCR amplicons can also be analyzed using dedicated microarrays such as the GeneChip Human Mitochondrial Resequencing Array 2.0 from Affymetrix. In general, these approaches are labor intensive, of low to moderate throughput, and expensive. Alternative methods simplified the workflow by targeting specifically the hypervariable regions, a small non-coding (but highly informative) portion of the mitochondrial genome. However, a limitation of Sanger sequencing is its lack of sensitivity, which restricts the identification to variants with an heteroplasmy frequency of over 10–20% [22]. This may be a major flaw for mutation carrier detection or in situations for which the extent of heteroplasmy of a given variant would be key for understanding the etiology of mtDNA related diseases (see above). Nowadays, more quantitative Next Generation Sequencing (NGS) technologies are replacing Sanger sequencing to characterize the PCR products. A recent method described for instance a panel of 161 tiling PCR products, covering the entire mtDNA, sequenced on a PGM (Ion Torrent Personal Genome Machine, Thermo Fischer) [23]. The most common methods have actually rather been focusing on the simplification of the PCR strategy, introducing long range PCRs covering the full mtDNA with only 1 to 3 amplicons [24–26]. The single amplicon PCR is a clear progress over the other PCR methods since it requires a single reaction well per sample, facilitating the setup and reducing the risk of handling errors. However, even when primer sequences are available from the literature, long range PCR can be complicated to establish as it may require the optimization of the buffer, as well as of the cycling conditions to achieve a robust and specific amplification. In addition, the PCR reaction can be impacted by the presence of a polymorphism in a primer binding site. This represents an important potential issue when considering that several thousands of human mtDNA variants are reported in the MITOMAP database, some being found at high frequency in the population [27]. Along the same line, it has been observed that NUMTs can be individual specific [19]. It may therefore happen that, for some individuals, primers could co-amplify NUMTs, again biasing the variant detection. For organisms less well characterized than human and mouse, such as the rat or the zebrafish which are widely used as model organisms in research, the absence of extensively validated PCR primers seriously complicates the design of PCR amplification of mtDNA. As an alternative to PCR amplification, mtDNA can be enriched by biochemical purification of the organelle [28, 29]. This approach can efficiently eliminate the nuclear DNA (carrying the NUMTs) but it requires relatively large amounts of fresh starting material, it is labor intensive, and its throughput is low. Another strategy, more in line with classical NGS methods, is to start from total DNA extracts but to sequence only a subset of the libraries which have been enriched for mtDNA by an hybridization-based capture using mtDNA-specific baits [8, 30]. Sequencing reads mapping the mitochondrial DNA are also found as by-product of whole genome or whole exome sequencing data, and have been used for several studies [7, 31–33]. However, such sequencing strategies have been designed for capturing the nuclear DNA information which is generally identical in all cell types and stable over time, and are therefore not optimal for extensive mtDNA research. A comprehensive overview of the different methods for high throughput sequencing of mtDNA has recently been published [34].

As an alternative to PCR, the amplification of mtDNA can be achieved using Rolling Circle Amplification (RCA, also known as MDA for Multiple Displacement Amplification). Random primed rolling circle amplification elegantly eliminates the PCR concerns described above since a universal setup fits all species, works without the need for technical optimization, and requires a single amplification reaction per sample. The circular nature of mtDNA (as opposed to the reaction priming for PCR) allows the specific and efficient enrichment of mtDNA versus nucDNA. Thanks to the high processivity and the strong strand displacement activity of the Phi29 polymerase used in the RCA reaction, a single priming event will indeed generate several copies of the mtDNA (circular template). In contrast, nucDNA (linear template) will only result in a 1:1 amplicon:template ratio (as illustrated in Additional file 1: Figure S3A). This approach has been previously used to amplify mtDNA [35, 36] but without further evaluating whether the product was suitable for low frequency variant detection. It is also worth mentioning that successful RCA amplification is achieved with input as low as 50 pg of total DNA (data not shown), as opposed to 100’s of ng of material used with PCR-based approaches.

Here, we describe MitoRS (for Mitochondrial DNA analysis by Rolling circle amplification and Sequencing), a novel mtDNA sequencing strategy for detecting mtDNA variants with high accuracy and sensitivity. The initial amplification of mtDNA takes advantage of the versatility of the RCA reaction. Libraries are then prepared at high throughput thanks to a tagmentation-based method, and sequenced to high depth by NGS. The analysis pipeline is tuned to detect low frequency heteroplasmic variants. The entire procedure is tailored to be high throughput and requires less than a day of work, even for a large number of samples. Extensive benchmarking experiments have been designed and are reported to demonstrate the actual performance of MitoRS. Detailed laboratory protocols, analysis pipeline, and benchmarking procedures are presented to allow straightforward implementation of the pipeline. We anticipate this method will promote the analysis of mtDNA polymorphisms.

## Results

### RCA validation for mitochondrial DNA enrichment

Accurate and sensitive analysis of mtDNA requires its enrichment over nucDNA. This step specifically limits the generation of undesired nucDNA sequencing data (sequencing “waste”) and allows working with low amount of starting material.

**Fig. 1:**
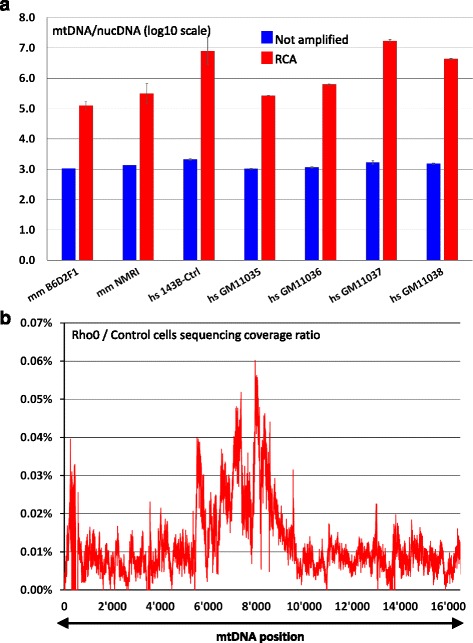
Rolling circle amplification of mitochondrial DNA. **a** Rolling circle amplification significantly enriches the mitochondrial DNA versus nucDNA. Absolute quantification by qPCR was performed to evaluate the ratio between mtDNA and nucDNA with or without RCA. Copy numbers are calculated from standard curves. Results are shown in log10 scale. Standard deviation are calculated from three independent qPCR experiments, on the same sample for the non-amplified material, and from three independent RCA reactions for the amplified samples. mm: mus musculus, hs: homo sapiens. **b** Nuclear reads contamination (NUMTs) does not affect MitoRS. Sequencing reads generated from a human control cell line (143-B) or its mitochondria-free derivative (143-B-Rho0) were mapped against the human mtDNA reference (rCRS, NC_012920.1). The ratio of the absolute coverage (reported by mpileup) between the Rho and control cell line was calculated for each position of the reference genome and plotted. The two datasets are generated from the same total number of reads (~15 million). Note that these samples had to be sequenced 10 times more than the usual procedure because of too few mapping reads for the Rho0 sample

In order to assess the actual level of mtDNA enrichment obtained by RCA, qPCR was performed to measure the relative amount of mtDNA versus nucDNA prior and after amplification. Total DNA extracted from different biological starting material was used for RCA: mouse liver, adherent human cells in culture, or immortalized B-lymphocyte (from the human DNA sample repository at the Coriell Institute for Medical Research). Depending on the sample, 100- to 10’000-fold RCA-dependent enrichment was achieved (Fig. 1a). We confirmed by restriction digest of the mouse DNA RCA products that the amplified DNA mainly consists of concatenated mtDNA (Additional file 1: Figure S3). Even with the lowest enrichment measured in mouse liver B6D2F1 liver extracts, we estimated a ratio of only one nucDNA molecule per 50’000 mtDNA molecules after RCA amplification. It is therefore very unlikely that NUMTs could interfere with variant detection following RCA enrichment.

We formally evaluated the extent of NUMTs contamination by comparing the sequencing results obtained from a cell line (human 143-B cells) and its derivative in which the mtDNA was depleted (143-B-Rho0 cells). For this purpose we applied the MitoRS method which is summarized (laboratory procedures and analysis pipeline) in the Additional file 2: Supporting Information and in the Additional file 3: Figure S1 and Additional file 4: Figure S2. All sequencing reads obtained from the Rho0 cells and mapping to the mtDNA should be originating from NUMTs contamination. We first confirmed by qPCR that the mtDNA was indeed reduced from ~ 2’000 copies (143-B cells) to the equivalent of less than 0.1 copy per cell in DNA extracted from these cell lines (143-B-Rho0 cells, data not shown). When analyzed with the MitoRS pipeline, the proportion of sequencing reads mapping to the mtDNA genome dramatically dropped from ~60% in the control sample to less than 0.05% in the Rho0 sample (the remaining reads mapping to nucDNA). The sequencing coverage ratio between the Rho0 and the control samples was calculated at each position of the mtDNA reference genome. The corresponding plot (Fig. 1b) highlights the fact that this NUMTs contamination is extremely low (below 0.06%). It is actually most probably the result of trace amount of mtDNA remaining in the Rho0 cells. This demonstrates that using the MitoRS experimental settings, NUMTs are not interfering with genuine mtDNA variant detection, even for low frequency heteroplasmy.

### RCA application for low frequency variant detection

RCA is a widely used technology for whole genome amplification, in the context of genotyping experiments for instance. The low error rate of the Phi29 polymerase (estimated to be in the 10^-6^ range [37]) has only minimal consequences when investigating normal diploid genomes for which a given variant will have a frequency of 0, 50 or 100%. The situation is different when investigating low frequency heteroplasmy as in the case of viral DNA, cancer cell nuclear DNA, or mtDNA analysis for example.

We therefore considered important to carefully evaluate the RCA-induced error by sequencing libraries generated through our pipeline, including or not the RCA step. Plasmid DNA was selected as the ideal starting material for this purpose because it can be produced at high concentration, to high purity, and is virtually clonal. This allows the generation of a control sequencing library directly from the crude starting DNA, without any pre-amplification step. We preferred plasmid DNA over PCR products because of its circular nature, similar to mtDNA. RCA reactions were seeded with approximately 5 million plasmid molecules (estimated from fluorescent-based DNA quantification) spiked into 5 ng of total mouse liver total DNA to mimic mtDNA amplification conditions. For the non-amplified conditions, the library was generated directly from crude plasmid DNA.

#### RCA does not introduce a coverage bias

**Fig. 2:**
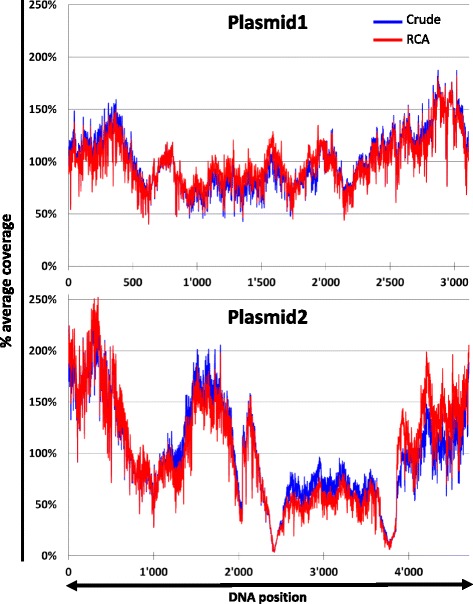
RCA does not introduce coverage biases. Sequencing reads were generated from two plasmid DNA with or without the RCA step. The relative coverage reported by mpileup was plotted against each position of the reference genome. A single replicate is presented though the exact same patterns were observed for independent triplicates. *Top panel*: plasmid1, *bottom panel*: plasmid2

As shown in Fig. 2, the sequencing coverage obtained from the crude and the RCA treated samples are nearly perfectly overlapping, indicating that RCA does neither over- nor under-amplify specific sequences.

Unlike Plasmid1, the coverage over the reference genome for Plasmid2 is not homogenous. This profile is similar with or without RCA, demonstrating that this variation is not the consequence of RCA. The origin of this phenomenon is most probably the result of biases introduced by the subsequent steps in the process such as the tagmentation, the PCR amplification of the library, the clustering, or the sequencing. This hypothesis is consistent with coverage differences commonly observed when comparing sequencing libraries generated with a normal and a PCR free method (our observations, data not shown).

#### RCA does not introduce technical variability

Each plasmid DNA was run as four independent replicates with or without RCA. These technical replicates are performed from the same plasmid DNA preparations. The technical reproducibility of the variant frequency call was evaluated by calculating the standard deviation within the four replicates.

This analysis shows that there is literally no variation for the two non-amplified plasmids, confirming that the library preparation and the sequencing procedures are extremely robust (Additional file 5: Figure S4A). Following RCA, we detected a slight albeit very low background noise with only few positions having a standard deviation over 0.1% (the VarScan variant detection threshold). The two positions with the highest standard deviation (>0.2%) can actually be fully explained by the presence of a high frequency variant detected in the plasmid DNA (see below).

#### RCA is accurate

We next evaluated whether RCA introduces errors in the template DNA to be sequenced, which would reduce the sensitivity and accuracy of the approach. To this end, we calculated the difference in the frequency of detected variants between the amplified and the non-amplified material for each position of the reference genome. Here we consider separately Single Nucleotide Variants (SNV) and Insertion/Deletion (Indel) events. Only few positions could actually be identified, and most of them with only minor differences.

**Fig. 3:**
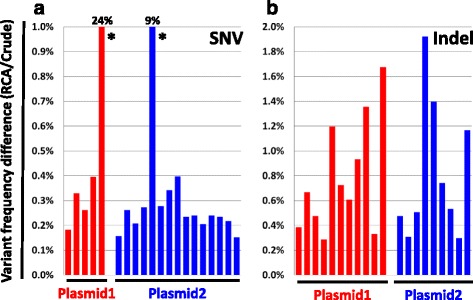
RCA does not introduce sequencing errors. The difference in absolute frequencies between the non-amplified samples and the RCA samples was computed for each single position of the reference genome. The positions with a non-null difference are plotted as a bar. **a** SNV and **b** indels are plotted in two different graphs. The two non-concordant positions from the SNV panel resulting from unspecific RCA amplification are marked with a star (see the Additional file 2: Supporting Information). The calculation was made as the average frequency within the four sample replicates. Only passing filter variants were considered. *Left panel*: SNV, *right panel*: indels

The high accuracy of the RCA amplification was demonstrated by the very low difference in the frequencies of SNV (below 0.4%, see Fig. 3a). The two discordant SNV positions (marked with a star in Fig. 3a) can conclusively be explained by the presence of contaminating nonspecific plasmid DNA reads originating from the Phi29 enzyme preparation (see the Additional file 2: Supporting Information). Importantly, such contamination is not a concern when analyzing mtDNA.

Small indels (maximum observed size of 2 nucleotides in this test) were also quantified accurately albeit with slightly lower precision than SNV (differences up to 2%, see Fig. 3b). This higher error rate for indels may be the result of Phi29 dependent proofreading activity [37]. In addition, slight inaccuracy for indel frequency evaluation is not unexpected because precise indel quantification may require local sequencing read realignment to account for unavoidable sequencing reads mapping errors.

Taken together, these data demonstrate that the RCA allows a robust amplification of mtDNA with very limited introduction of errors. It is therefore fully compatible with low frequency mtDNA variant analysis by NGS, considering a lower threshold in the 0.5% range for SNV and 2% for indels.

### Benchmarking of MitoRS accuracy and sensitivity

We next assessed the accuracy and sensitivity of the MitoRS pipeline to quantify heteroplasmy frequency. DNA was prepared from the livers of two mouse strains (B6D2F1 and NMRI) whose mtDNA differ at more than 90 positions, offering many distinct sequence contexts to benchmark the pipeline. Variant identification from pure B6D2F1 and NMRI DNA extracts is detailed in the Additional file 2: Supporting Information. The two total DNA preparations were mixed at various ratios and analyzed. A total of 12 mixtures of NMRI/B6D2F1 DNA at ratios ranging from 0 to 100% were assayed. For each ratio, three independent RCA reactions were performed. Total amount of DNA (5 ng) and therefore the mtDNA copy number (~5 millions) was kept constant for each ratio. The exact mixture ratio was precisely calibrated from the frequencies obtained for the 50/50 ratio (see the Additional file 2). The average coverage was around 3’000X.

#### Benchmarking SNV detection

**Fig. 4:**
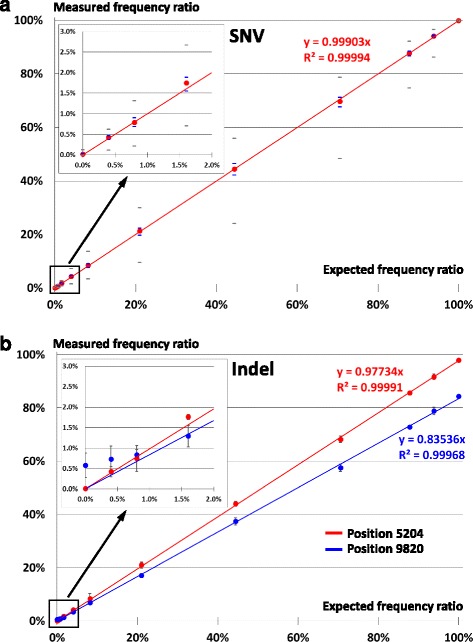
Benchmarking MitoRS accuracy and sensitivity. **a** SNV detection is accurate over the whole range of frequencies. Total DNA extracted from two mouse strains was mixed at different ratios and run through the pipeline. The measured frequency of the 88 homoplasmic SNV distinguishing the mtDNA from the two strains are plotted versus the calculated ratio from the input mixture. *Red dots* correspond to the mean frequency calculated from the 88 variants, internal *blue bars* show the 25th and 75th quartile, and extremal *grey bars* the minimum and maximum variant frequency observed for a given ratio. The correlation factor and the slope of the linear regression are shown on the graph. Three independent input mixtures were run for each theoretical ratio. The insert panel is a zoom on the low frequency ratios. **b** Indels analysis is also accurate. Same graph as in A., but considering only the two indel positions distinguishing the mtDNA from the two strains. The insert panel is a zoom on the low frequency ratios. Results are calculated as the average and standard deviation of the three independent RCA reactions performed for each ratio

We first focused on the 88 homoplasmic SNV that are distinct between the two mouse strains mtDNA (see the Additional file 2: Supporting Information). Results are presented with box plots to report the behavior per variant (Additional file 6: Figure S5A) as well as with a general plot to present a global picture of MitoRS performance (Fig. 4a).

When considering the median values of measured frequencies compared to the expected frequencies (based on the known mixture ratio), we observed a very high correlation (Pearson coefficient = 0.99994) with a slope very close to 1 (slope = 0.99903). This demonstrates that our pipeline faithfully delivers frequencies of heteroplasmy measured for any variant very close to its actual value. Importantly, this also holds true for the lowest frequencies we assayed (<0.5%, see Fig. 4a insert panel).

The 25 and 75% quartiles are always close to the median, indicating that there are only few outliers amongst the 88 individual SNV. A closer inspection of individual ratios and SNV (see Additional file 6: Figure S5 and Additional file 7) reveals that only few SNV are actually detected with lower accuracy and that this behavior seems to be position dependent. This suggests that the sequence context can influence the quantification of SNV frequency (binomial regression, *p*-value = 5.6 × 10^-9^). We did not observe any relationship between lower accuracy quantification and the coverage level or the sequence itself. The only positive correlation we identified was the surrounding variant density (variant density in a 300 bp window since we use 150 cycles Illumina reads) centered on the variant of interest, (Poisson regression, *p*-value = 2 × 10^-16^). Accordingly, the height positions for which the frequency underestimation is the highest could actually be clustered into two regions carrying four variants in a window of ≈ 50 nucleotides (see Additional file 6: Figure S5B and Additional file 7). We hypothesize that a higher variant density may impair proper sequencing read mapping as a read with many variants will not have a mapping score as good as a read perfectly matching the reference. Note that even with the SNV deviating the most from the expected frequency (position 15’588), the variant is detected at a sensitivity below 1% with accuracy (~2-fold underestimation for the lowest frequencies).

The result presented above were focused on the 88 expected SNV, not taking in account the VarScan default *p*-value threshold of 0.001. If this filter is applied, less than 30% of the 88 SNV are actually detected for the minimum theoretical 0.4% ratio. It increases to 80% for the 0.8% ratio and all 88 variants are detected for any higher ratio tested (i.e 1.6% and above). This means that there is a gray zone in which some genuine SNV are not recognized as variants by VarScan (false negative positions). Importantly, for any of the mixture ratio tested, no unexpected SNV (false positive) could be identified at this *p*-value (<0.001). This gray zone is therefore not affected by false positive SNV. Lowering the *p*-value stringency would reduce the number of false negative (ignored genuine SNV) but concomitantly result in some false positive SNV (unexpected variants). We also tested a 10-fold increase in the sequencing depth (average mtDNA coverage >30’000X) and observed the same trend, i.e. an increased sensitivity balanced by a higher background (data not shown). Based on the large sequence context diversity analyzed here (88 distinct SNV), we decided to set the MitoRS SNV detection frequency threshold at 1%. Nevertheless, the actual lower limit of detection to consider depends on the application and the biological question (tradeoff between false positive and false negative rate).

#### Benchmarking indel detection

Indel analysis was performed as for SNV but exploiting only the two positions for which indels were identified: position 5’204 (+G, 98% in NMRI) and position 9’820 (+AA, 84% in NMRI) (see the Additional file 2: Supporting Information). Note that position 9’820 is in a potentially complex context given the AA insertion lies in front of a stretch of eight As.

As for SNV, we observed a very high correlation across the different ratios which were analyzed (Fig. 4b). It is interesting to note that the slope is different from 1 (0.977 and 0.835 for positions 5’204 and 9’820 respectively). This is perfectly in line with the fact that both variants are not homoplasmic but are found in 98 and 84% of the mtDNA molecules respectively (see the Additional file 2: Supporting Information). This demonstrates that our pipeline can accurately and robustly determine indel frequencies.

In terms of sensitivity threshold, we have previously shown that the RCA induces errors as high as 2%. Here, a G insertion at position 5’204 is still accurately detected at ratios as low as 0.4%, whereas the background level is in the range of 1% for the more complex 9’820 position. Nevertheless, the VarScan *p*-value threshold (*p*-value < 0.001) restricts the analysis to mixture ratio above 1.6% so that the two indels are not considered as false negative. Moreover, data from the pure NMRI and B6D2F1 mtDNA runs revealed 5 false positive indels (passing the *p*-value filter) with frequencies as high as ~7% for position 5’171 (see the Additional file 2: Supporting Information and the Additional file 7). These are however associated with particularly complex sequence contexts. As a consequence, although we demonstrated that indels can be accurately quantified at frequencies as low as 0.5%, we considered that the relatively high level of false positive imposes MitoRS a conservative minimal threshold of 10%.

### Application of MitoRS to mtDNA heritability analysis

#### Analysis strategy

To apply the MitoRS pipeline, we investigated the heritability of mtDNA variations within the full CEPH families 1463 and 884. These large families offer the possibility to study the transmission of mtDNA variants over three generations. The full mtDNA of 17 (family 1463) and 18 (family 884) individuals were sequenced and analyzed. Data interpretation presented below could be performed using simple calculation, sorting, and filtering tools applied to the final output csv file generated.

Based on the benchmarking performed on mouse mtDNA (see above), variants were filtered for a minimum frequency of 1% for SNV and 10% for indels. Most of the mtDNA could be successfully included in the analysis, except few low complexity positions, mostly around the 310 C-stretch (labeled with “EXCLUDE”, see details in the Additional file 2: Supporting Information). For simplicity, we also chose not to report variants flagged for their mapping coverage below 10% of the average coverage (marked with “WARNING”, see details in the Additional file 2: Supporting Information). A table summarizing positions with identified variants can been found in the Additional file 8 and Additional file 9).

**Fig. 5:**
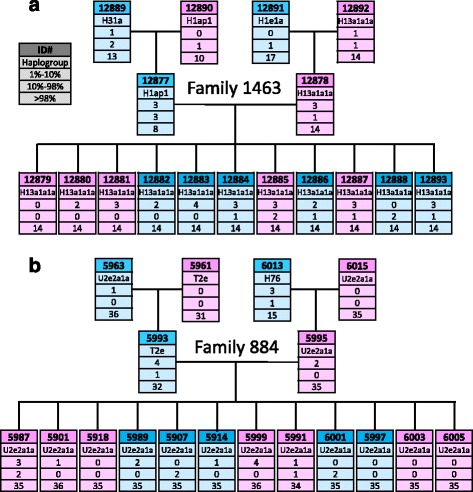
Mitochondrial DNA variant heritability. The DNA from the 17 and 18 members of the CEPH families 1463 (**a**) and 884 (**b**), respectively, were analyzed with MitoRS. Each haplotype was determined. Mitochondrial DNA variants were classified into three categories based on their frequency status: homoplasmic (>98%), high frequency heteroplasmy (between 10 and 98%) and low frequency heteroplasmy (between 1 and 10%)

For an easy visual overview, identified variants were separated into 3 classes based on their frequency, and presented as a pedigree tree (Fig. 5). The 3 classes of variants are: homoplasmic (frequency > 98%), high frequency heteroplasmy (between 10 and 98%) and low frequency heteroplasmy (between 1 and 10%). Each haplogroup was assigned by submitting the generated consensus fastA file to the HaploFind tool [38]. The Haplofind outputs can be found in Additional file 10: Table S2 and Additional file 8 and Additional file 9. As expected, all children share their mother’s haplogroup. This is actually the consequence of most homoplasmic variants being shared, which is not the case for heteroplasmic variants (see below). Note that some variants are at the limit of the 10% coverage threshold and may not always be reported in the pedigree tree. This is for instance the case for the homoplasmic variants at position 263 in the family 884.

#### Paternal mtDNA transmission

The dataset was screened to investigate putative low level of paternal mtDNA transmission. A comparable analysis has recently been performed by taking advantage of ultra-deep sequencing [39]. Despite the lower depth of our sequencing dataset, our approach has the advantage of being able to interrogate the full mtDNA genome. Importantly, a 10-fold increase in the coverage did not reveal additional variants in our dataset (data not shown). From the six fathers analyzed (three for family 1463 and three for family 884), no paternal specific variant, i.e. not also found in the mother’s mtDNA, could be identified in the offspring (summarized in Table 1 for family 1463, and Additional file 8 and Additional file 9 for complete results). This does not only hold true for homoplasmic position, as expected, but for heteroplasmic variants as well. Taken together, our results further land support against the transmission of mtDNA from the male in humans.

**Table 1:**
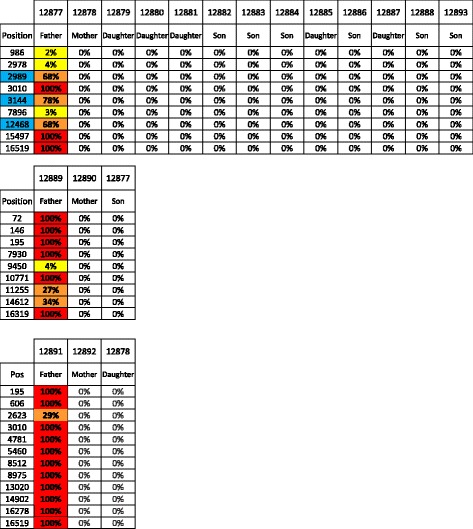
No evidence for father’s mtDNA transmission

Variants specific for the father (i.e. not also present in the mother) are shown. For easier visualization, homoplasmic variants passing filters are highlighted in red, high frequency heteroplasmy in orange, low frequency heteroplasmy in yellow, and positions not passing filters are left in blank. Each variant is ordered by lane and identified by its position (rCRS numbering). The positions highlighted in blue were verified by Sanger sequencing

#### Maternal mtDNA transmission

Mitochondrial DNA transmission from the mother was also scrutinized (summarized in Table 2 for family 1463, and Additional file 8 and Additional file 9 for complete results).

**Table 2:**
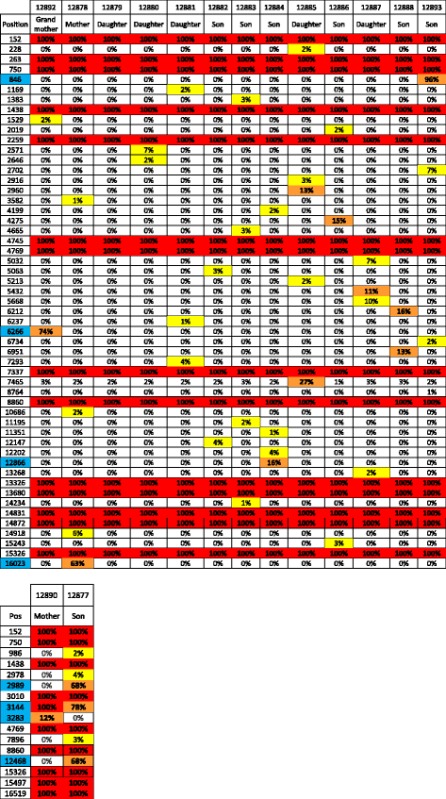
Transmission of variants from the mother’s mtDNA

Variant identified in the mothers or in the children are shown. Mothers #128892 and #12878 are shown in the same table to account for the three generations inheritance. The color code is the same as in Table 1. Each variant is ordered by lane and identified by its position (rCRS numbering). The positions highlighted in blue were verified by Sanger sequencing

We observe that homoplasmic variant transmission is the general rule since most homoplasmic positions are found identical between mother and children (and grandchildren). There are however several individual specific cases of positions shifting from maternal homoplasmy (<1% or > 98%) to children high frequency heteroplasmy (between 10% and 98%) in both families. Some of these frequency changes were of very high amplitude such as for instance positions 846 (93% in son #12893 versus 0% in his mother #12878 and his grandmother #12892) or positions 2’989 and 12’468 (both found at 68% in son #12877 versus 0% in his mother #12890). Maternal heteroplasmic variant transmission could only be analyzed from 3 positions. None of these were actually inherited: position 3’283 (12% in mother #12890 versus 0% in her son #12877), position 6’266 (74% in grandmother #12892 versus 0% in all other family members) and position 16’023 (63% in mother #12878 versus 0% in all other family members). The height positions showing the highest frequency difference were validated by Sanger sequencing (see the chromatograms and the quantification results in the Additional file 11). For instance, position 846 A to G polymorphism lies in the 12S ribosomal RNA, position 6’266 A to G is a silent mutation in the COX1 gene, and position 16’023 G to A is the last nucleotide of the tRNA proline gene. None of these 3 mutations are reported in the MITOMAP variant list [27].

Although these findings are not in complete disagreement with the bottleneck hypothesis for mtDNA transmission [11], the extent of appearance/disappearance of high frequency variants is unexpected. It may be the consequence of de novo somatic mtDNA mutation, a phenomenon reported in many studies involving mtDNA variant heredity and/or tissue specificity analysis [8, 10, 40]. Accordingly, sequencing additional tissues would help understanding whether what we observe is blood cell specific somatic mutations (DNA samples have been extracted from immortalized lymphoblastoid cell lines). The co-occurrence of the variants in several tissues would at the opposite strongly suggest that they have been inherited from the mother. The shifts may also be the result of ex vivo genetic drifts since these DNA are originating from blood cells collected in the 80s, immortalized and having undergone extensive passages. Given this behavior is observed for both families 884 and 1463, our results question whether such DNA collections represent an appropriate source of material for studying mtDNA polymorphism.

## Discussion

### Method design

One of the pillar of MitoRS is the rolling circle amplification of mtDNA. We selected RCA rather than PCR because 1) it does not require primer design; 2) it amplifies preferentially the circular mtDNA over linear nuclear DNA, thereby reducing the NUMTS contamination; 3) it involves a single reaction per sample, and 4) it is easy to setup. The main difficulty of a PCR approach lies in the design of PCR primers which should be efficient and specific, but tolerant to polymorphisms. Designing a PCR primer first requires a reference sequence of quality. This is the case for a growing number of species though many new full mtDNA genomes are still being published [41, 42]. The human mtDNA is very well characterized with more than 32’000 full length mtDNA sequences compiled into the MITOMAP database [27]. This large number of sequences actually raises the question of the potential impact of mtDNA variants on PCR. The PCR reaction can indeed be influenced by the presence of a polymorphism in a primer binding site since it can reduce amplification efficiency, or even prevent the amplification of a whole mtDNA (sub)population. In both cases, this would lead to erroneous variant frequency quantification in a situation of heteroplasmy. To note, most of the PCR primers described in the literature actually takes in account the known polymorphisms, though some of them maybe questionable. Overall, RCA presents the great advantage of offering a universal and technically simple approach as compared to PCR. Combined with the tagmentation-based library generation and the magnetic bead-based DNA purification, the entire MitoRS procedure is tailored to be high throughput and requires less than a day of work. To the best of our knowledge, this represents a significant improvement compared to classical PCR-based or capture-based methods previously described in the literature.

Efficient DNA amplification requires high quality DNA templates. For instance, DNA nicks or abasic sites can be a major obstacle for faithful RCA reactions, and it has been previously reported that low quality samples (such as some forensic samples) may be challenging to amplify by RCA [43]. This constraint is however not specific to RCA as it also impacts applications involving long range PCR amplification. As a consequence, heavily degraded mtDNA may only be amenable to focused analysis (i.e. small regions covered by short PCR products [23]), whereas extensive full length analysis, as described by this method and others, requires good quality template.

Eventually, standard tools developed for the analysis of NGS data were combined and parametrized to meet the special requirements of mtDNA sequencing datasets, which involve a small reference genome, an extremely deep coverage, and the presence of low frequency heteroplasmic variants. Importantly, variants close to the origin can readily be detected because the circular nature of mtDNA is taken into consideration. The tools we used are open access and the script parameters are described, simplifying the pipeline deployment in any laboratory with access to NGS technology. Note that read mapping by BWA is performed on the mitochondrial DNA only and not on the nuclear genome, which reduces the computation effort required. The final csv-based reporting file was specifically designed to simplify data handling and sample-to-sample comparison. The compilation of variant frequency over multiple samples and positions can then be performed by the end user using spreadsheet management tools, and without the need for advanced bioinformatics tools. Classical VarScan outputs are also available for more advanced analysis.

### Method benchmarking

With MitoRS, our objective is to present a trustable method which could easily be implemented. We therefore performed an extensive set of benchmarking experiments in order to describe the performances and the limitations of the pipeline.

The low error rate of the Phi29 polymerase is not problematic for diploid genome analysis, while it could be critical for more demanding applications which require the detection of low frequency variants. By comparing sequencing results from RCA treated to non-amplified plasmid DNA, we demonstrate that the RCA is extremely accurate with only few differences identified with a maximum frequency below 0.4% for SNV and 2% for indels. To the best of our knowledge similar performance benchmarking is not available for mtDNA amplification methods based on long range PCR.

A deeper benchmarking of MitoRS was subsequently performed with mtDNA, the actual target of the method. To this end, we analyzed multiple ratio mixtures of two mtDNA sources differing at ~90 homoplasmic positions, representing a large diversity of sequence context surrounding variants. The accuracy obtained is extremely high since, for SNV or indels, the frequency of variants measured experimentally was perfectly corresponding to the expected frequency (Pearson coefficient > 0.999).

We demonstrate the high sensitivity of MitoRS as we could detect all the expected SNV at a frequency as low as 0.4%. However, these variants are actually to be considered as “not detected” because of a VarScan *p*-value above the 0.001 threshold. For this reason, we defined a conservative minimal frequency threshold for the detection of SNV heteroplasmy at 1%. This performance was not improved by increasing ten-fold the sequencing depth. With this dataset, the limit of detection can indeed be lowered below a frequency of 0.5%, but this is achieved at the expense of an increased number of false positive SNV, resulting in the same final threshold for the detection of true positives. Reaching a higher level of sensitivity rather requires alternative more accurate sequencing strategies involving several sequencing rounds on the same molecule, for example with duplex sequencing [13] or circular sequencing [44]. The long read based circular consensus sequencing strategy offered by the Pacific Biosciences sequencing platforms may yet be an alternative suitable approach to this end.

The detection of indels in mouse mtDNA was also accurate in the frequency range of 1%. The analysis suffers however from a higher false positive background noise, which imposes a conservative minimal frequency threshold at 10%. This high false positive rate mainly results from the difficulty to properly align sequencing reads in the context of homopolymeric repeats. A potential approach to improve the precision in the quantification of the short indels frequency may be to perform a local realignment around the problematic positions.

Large indels are not considered in this analysis. By lengthening or shortening the reference sequence (therefore artificially simulating deletions or insertions), we observed that large indels from few bases to several kb could be readily identified (data not shown). The difficulty does not actually lie in the detection of large indels, but rather in estimating the heteroplasmy level of truncated mtDNA populations. The quantification of large indels indeed requires a very homogenous coverage as described with an approach involving mtDNA enrichment from a single PCR product [26]. The precise quantification of low level heteroplasmy for large indels and/or multiple co-existing truncated mtDNA is a complex task for which NGS technology may not be the most appropriate tool.

In all the human samples we processed, the entire mtDNA genome could be analyzed with the exception of approximately ~15-nucleotides around the position 310 C-stretch. This difficulty has already been reported [40, 45] and this region is generally excluded from the analysis. In our pipeline, this position is automatically excluded (coverage below 1% of the average sample coverage), and Ns are actually populated in the output consensus sequence to prevent false positive variant reporting. There are also a few additional positions for which we identified variants but did not report them because they were flagged for low coverage (e.g. positions 263, 513, 16’182, and 16’188 in CEPH family 1463). Our strategy is actually to output the analysis results from all the reference genome positions. The different quality metrics available from the output files, together with the benchmarking results presented here leave the choice to the end user to decide what is considered as a reliable variant. One may for instance disregards indels, adjust the coverage filter, or consider a lower SNV threshold at 0.5%. Refer to the Additional file 2: Supporting Information for details on the procedure we used to handle low complexity regions.

In summary, MitoRS allows to call with high confidence variants of heteroplasmy frequencies as low as 1% for SNV, and 10% for indels, for more than 99% of the mitochondrial genome.

### MitoRS applications

We applied MitoRS to further investigate the heritability of mtDNA in humans by sequencing the full-length mtDNA from 35 samples with minimal laboratory workload and analysis efforts. The sensitivity and the accuracy achieved enabled us to exclude paternal mtDNA transmission, even at low frequency, confirming and strengthening previous findings [39]. We could also follow the heritability of mtDNA polymorphisms of maternal origin. We observed that the transmission of homoplasmic variant is the general rule even though several instances of high amplitude shifts between homoplasmy and high frequency heteroplasmy could be reported. With a single tissue and three generations analyzed, it is difficult to distinguish variant heritability from de novo somatic/ex vivo mutations. Here, it may therefore be more accurate to state for variant sharing rather than for variant inheritance. The MitoRS method could actually help clarifying this type of question by promoting large-scale studies on mtDNA variant inheritance.

Being based on RCA, MitoRS does not require prior knowledge of the target mtDNA sequence, as it is the case when PCR primers have to be designed. Mitochondrial DNA from poorly characterized organisms can therefore be easily sequenced and even screened for heteroplasmy. Importantly, this is not limited to the characterization of mtDNA but may also be applied to small to moderate size circular DNA such as viral DNA, or bacterial plasmid DNA.

Whole genome or whole exome sequencing datasets initially generated to study variants from the nuclear genome have been successfully exploited for the analysis of mtDNA variant [6, 7, 17, 31–33]. The very large and ever increasing public genotyping datasets (from WGS or exome sequencing) can therefore be a valuable source of information. However, mtDNA analysis studies should be able to capture tissue specificity and variability over multiple time points (see Li et al. [10] for instance). Together with the fact that the extent of NUMTs contamination from whole genome or exome sequencing datasets is not completely determined, it is unlikely that these public data would be adequate for extensive mtDNA research. To the best of our knowledge, the largest study fully dedicated to mtDNA analysis is based on a capture protocol. It includes nearly 2’000 samples (12 tissues from 157 individuals) with a variant frequency detection sensitivity threshold stated at 0.5% [10]. The ability to include such a large number samples and to work with such a low detection sensitivity was key for the authors to make ground breaking observations about mtDNA heteroplasmy (tissue specificity, active variant selection, influence of age and other parameters). Such throughput is however an exception in the field and is not accessible to most laboratories because of the large workload required to generate the sequencing libraries. The protocols proposed to date are indeed labor intensive in terms of library generation since they can involve a large number of steps such as multiple PCR reactions, DNA shearing, sequencing adaptors ligation, or capture-based enrichment for instance. MitoRS greatly reduces and simplifies these steps, making large-scale analysis more amenable and affordable. In terms of sequencing load, the results presented here were obtained with the equivalent of ~120 samples loaded per lane of a HiSeq flow cell, a high multiplexing rate which strongly reduces the cost per sample.

In numerous fields as for instance forensic science, haplogroup assignment, or population genetics, there is a growing interest in gaining discrimination power by analyzing the full-length mtDNA as opposed to limit the study to the hypervariable regions or to a single mitochondrial gene. In addition, being able to accurately detect and quantify low frequency heteroplasmy is highly relevant. Most observed heteroplasmic positions indeed have frequencies below 10% [6, 7, 10], probably explaining why homoplasmy was thought to be the rule before NGS was deployed. It is therefore crucial to be as sensitive as possible in order to obtain the most comprehensive and accurate assessment of heteroplasmy. With MitoRS, the easy access to increased sensitivity makes possible the follow up of low frequency variants over multiple tissues and time points, for example in the course of ageing studies. Similarly, it can be used as tool to control mitochondrial genome integrity in the context of induced pluripotent stem cells (iPSC) generation since it has been recently demonstrated that some reprogrammed clones may accumulate deleterious variants present only at low frequency in the donor cells [12, 18]. By allowing a systematic variant analysis, MitoRS may also help in better understanding the biological relevance of the increasing list of mitochondria-encoded short peptides described in the literature [3]. Furthermore, mitochondrial dysfunction has been linked to cancer [46] though the exact role of mtDNA itself remains unclear [32, 47]. In the heterogeneous cancer tissue, the excess of non-polymorphic DNA may mask the presence of a variant of interest. The problem of heteroplasmy is in this context also a concern for nucDNA somatic mutations, but the question of sensitivity is further exacerbated in the case of mtDNA given that heteroplasmy can already be present at baseline under normal (not cancer-affected) conditions. Mitochondrial DNA analysis must therefore be able to detect very low frequency variants. The lower limit of detection becomes particularly key when mtDNA variants are exploited as a biomarker for early detection of tumor or prediction of relapse for instance [8, 24].

## Conclusions

We describe MitoRS, a novel mitochondrial DNA variant analysis method. The main particularity of MitoRS is to use Rolling Circle amplification as an alternative to PCR for the enrichment of mtDNA. Compared to PCR, this approach enables a universal (not species-specific, and insensitive to NUMTs and to mtDNA polymorphism) and simpler reaction setup, and opens the way for larger scale studies. Importantly, this simplification is not achieved at the expense of quality since the robustness, accuracy and sensitivity performance we obtain are similar or outperform the methods classically described in the field.

We anticipate that MitoRS will advance the more systematic analysis of mtDNA and will help to assess the contribution of mtDNA heteroplasmy to the development of metabolic disorders, cognitive decline, cancer, or age-dependent loss of tissue function. In addition, given the very low requirement of input material, our method is an important step towards the investigation of mtDNA heteroplasmy in single cells.

## Methods

### DNA source

Human DNA from the CEPH families 884 and 1463 was obtained from the NIGMS Human Genetic Cell Repository at the Coriell Institute for Medical Research (extraction performed from immortalized white blood cells). For the 143-B cell line and its mitochondria depleted counterpart (143-B-Rho0), DNA was obtained from RhoZero Technologies (Uettingen, Germany). Unused livers from mice sacrificed for other experimental purpose were obtained from the EPFL animal facility (authorization number VD 1832.3 Lausanne, Switzerland). Livers were minced with scissors and homogenized in Sucrose buffer (250 mM Sucrose, 5 mM Hepes, 2 mM EGTA, pH 7.4). DNA was extracted from the homogenate using the DNeasy Blood & Tissue Kit (Qiagen).

### qPCR quantification of mtDNA

Quantitative PCR was performed from 5 ng total DNA or 15 ng RCA product with the LightCycler1536 DNA Green Master on a LightCycler 480 Instrument II (Roche). Two nuclear genes and two mitochondrial genes were measured, both for mouse and human samples (see primer and probe sequences in Additional file 12: Table S3). Absolute quantification was performed thanks to a standard curve (E1 to E9 copies per microliter) included in each run. The standard curve was generated by cloning the four PCR amplicons in plasmid DNA. Plasmid DNA was then precisely quantified by fluorimetry (Quant-iT Picogreen dsDNA assay, Thermo Fischer) and pooled at an equimolar ratio.

### MitoRS library generation and sequencing

The RCA is performed in 96-multiwell plate from 5 ng DNA with the REPLI-g Mitochondrial DNA Kit from Qiagen, strictly following the manufacturer’s recommendations. Irrespective of the source of DNA (human, mouse or plasmid), the supplied human mtDNA specific oligonucleotides are used (with no effect on yield, data not shown). The reaction is purified with Ampure beads (Beckman) at a 0.5X ratio. Purified DNA is quantified by fluorimetry (Quant-iT Picogreen dsDNA assay, Thermo Fischer) and its size estimated with the Tapestation (Genomic DNA ScreenTape, Agilent). DNA is normalized and 1 ng used to generate a sequencing library with the Nextera XT kit (Illumina) strictly following the manufacturer’s recommendations, followed by a final Ampure bead purification at a 0.6X ratio. Purified DNA is quantified by fluorimetry and the library quality monitored with the DNA High Sensitivity Reagent kit on a LabChip GX (Perkin Elmer). Libraries are pooled equimolar and run at 6 picomolar (spiked with 3% PhiX) for a paired end rapid sequencing run of 2 x 150 cycles on a HiSeq 2500 (Rapid SBS kit v1, Illumina). The number of sample loaded per flow cell was variable but, when necessary, sequencing data were all downsized to an equivalent of ~120 samples loaded per HiSeq flow cell lane (corresponding to ~1.5 million reads per sample). An overview of the laboratory procedures is shown in Additional file 3: Figure S1.

### Analysis

The fastQ files are first aligned with the Burrows-Wheeler Aligner (BWA-MEM version 0.7.4, [48]) using default parameters (v = 1 and t = 5). The mtDNA reference sequences used are the rCRS sequence (NC_012920.1) for human, and the NC_005089 for mouse, respectively. The resulting bam files are then computed with mpileup (Samtools v0.1.19) to filter for high quality bases and alignment (Q = 35, q = 50 and C = 50). Variant are subsequently called with VarScan2 (version 2.3.6) using default parameters (*p*-value < 0.001 and min-var-freq = 0.1%). The classical VarScan commands mpileup2snp and mpileup2indel were run to identify single nucleotide variants and small indels respectively. We also used the mpileup2cns command in order to generate an output file including all positions of the reference genome. A relative coverage was computed by normalizing the absolute coverage at a given position by the average coverage measured over the entire reference genome for the same sample. This relative coverage is flagged as “GOOD”, “WARNING”, and “EXCLUDE” based on thresholds at 10 and 1%. When a given position is flagged as “EXCLUDE”, the reported consensus is an N and the variant frequency is set to 0% to prevent the pipeline from reporting false positive variants. An additional column is added to the mpileup2cns output. It is filled with a per position sequence output in which only the major allele is reported (taking in account all quality filters mentioned), or an N in case variants analysis fell outside the quality criteria. This column is used to report a major haplotype in a fastA format. The overall procedure is performed twice, once with the original reference sequence and the second time with a reference from which the +1 position is shifted approximately to the center of the reference sequence (“shifted reference”). Refer to the Additional file 2: Supporting Information for further details. The two output files are subsequently merged, keeping only the per position data from the file for which the coverage is the highest. The script overview is available in Additional file 4: Figure S2.

Haplogroup assignment is performed with the HAPLOFIND tool [38]. The input used is the consensus fastA file generated by the pipeline.

### Sanger sequencing validation of human high frequency heteroplasmy variants

Five regions encompassing high frequency heteroplasmy variants were PCR amplified: region A (position 652 to 934), region B (position 2865 to 3457), region C (position 6’129 to 6’467), region D (position 12’359 to 12’990), and region E position (15’873 to 16’295). Attention was given to avoid primer binding site overlapping a polymorphic position identified by MitoRS. Primers are listed in Additional file 12: Table S3. PCR was performed for 35 cycles from 30 ng input DNA using the Hot Start HiFi polymerase (Kapa Biosystems). PCR products were purified with Ampure beads (Beckman) at a 1.8X ratio. Sanger sequencing was performed with the big Dye terminator version 3.1 (Thermo Fischer) using both forward and reverse PCR primers. The level of heteroplasmy at the positions of interest was evaluated with the ab1 Peak Reporter software (Thermo Fischer, https://apps.thermofisher.com/ab1peakreporter/). The quantification results are presented in in the Additional file 11.

## Additional files

Additional file 1: Figure S3. RCA enriches circular versus linear templates. A. Principle of circular DNA enrichment. A single priming event will generate several concatenated copies of a circular template. At the opposite, a single copy will be amplified if the template DNA is linear. B. The RCA amplified material is mostly mtDNA. Digestion of the mouse DNA RCA product with the SpeI restriction endonuclease results in the expected mtDNA digestion product with only low amount of undigested DNA left. SpeI restriction digest is expected to result in four fragments for the B6D2F1 strain (7’398, 3’759, 3’105, and 2’035 bp) and five fragments for the NMRI strain (7’398, 3’105, 2’150, 2’037, and 1609 bp). Ladder sizes imprecision is in accordance with the Agilent Genomic DNA ScreenTape specifications. (PPTX 133 kb)

Additional file 2: Supporting Information. (DOCX 56 kb)

Additional file 3: Figure S1. Overview of the MitoRS wet lab method. Total DNA (5 ng) is amplified by RCA. Sequencing libraries are subsequently generated using the Nextera XT kit from Illumina, starting from 1 ng amplification product. Libraries are pooled at equimolar ratios and sequenced on a HiSeq 2500 (Illumina) using rapid mode for a paired-end run of 2 x 150 cycles. (PPTX 40 kb)

Additional file 4: Figure S2. Overview of the MitoRS analysis methods. FastQ files are aligned with BWA to the original, and an origin-shifted version, of the DNA reference sequence (refer to Additional file 2 for details). Variant frequency is evaluated by samtools mpileup. Low frequency variants are finally identified using VarScan 2. Datasets generated from both the original reference and the shifted reference are merged, keeping only the per position values from the dataset with the highest coverage. Data are further processed to generate 1) a csv file summarizing the sequencing results observed at each individual mitochondrial DNA position and 2) a fastA file representing the corresponding consensus sequence. More details can be found in the Methods section. (PPTX 264 kb)

Additional file 5: Figure S4. The RCA procedure is robust. A. RCA does not introduce technical variability. Each individual plasmid DNA was run as four independent replicates for both conditions (with or without RCA). The technical reproducibility of the variant frequency call was evaluated by calculating the standard deviation within the four replicates. Value are plotted for each individual position of the reference sequence, for the two plasmids sequenced. Data are directly extracted from mpileup analysis applying a VarScan p-value threshold of 0.001. Left panels: plasmid 1, right panels: plasmid 2, top panels: crude plasmid DNA (no RCA amplification), bottom panels: RCA amplified plasmid DNA. B. Origin of the nonspecific RCA products. The sequences surrounding the unique position for which a large difference was observed between the crude plasmid DNAs and the RCA products were aligned. The sequence obtained from contaminating NTC reads is shown. The common (between the two plasmids) non-accurate position is boxed (“G” in the NTC, “A” in the two plasmids). This region is part of the plasmid DNA origin of replication. (PPTX 178 kb)

Additional file 6: Figure S5. Mouse SNV frequencies are homogenous within the 88 position analyzed. A. Individual SNV frequency. For the 12 mouse mtDNA mixture ratios tested, the frequencies measured for each SNV (88 in total) were plotted and summarized as a boxplot. The boxplot whiskers highlight the extreme values (min and max). Note that the scale is different for each plot. The eight positions showing the highest frequency underestimation are highlighted in blue. These data were used to build the Fig. 4. The raw data are available from the Additional file 7. B. The eight SNV with underestimated frequency are located into two dense clusters. For each 88 SNV, the deviation from the theoretical frequency was calculated and plotted versus their position in the mitochondrial genome. The eight positions showing a systematic frequency underestimation highlighted in A. are also shown in blue. They cluster into two very short genomic regions. (PPTX 484 kb)

Additional file 7: MitoRS output file for the pure and mixed B6D2F1 and NMRI samples sequenced for the benchmark of mitoRS accuracy and sensitivity. Tab1: Raw MitoRS output for the mouse mtDNA mixtures. Samples are named based on the theoretical mixture ratio (% B6D2F1 DNA) and replicates (A, B, or C) for a total of 36 samples analyzed. Column headers are detailed in the Additional files 14: Table S1 legend. Data from the 36 samples are populated in consecutive columns. Tab2: List of variants identified from unmixed mouse B6D2F1 and NMRI mtDNA. All NMRI and B6D2F1 mtDNA positions for which a variant was identified compared to the mouse mtDNA reference are listed. Pos: position in the mtDNA, Freq: variant frequency, SD: standard deviation within the three replicates, *p*-value: VarScan calculated *p*-value, Ref: Nucleotide from the reference genome, Var: Alternative nucleotide identified by VarScan, Cons: Consensus nucleotide kept by VarScan, Type: type of variant (highlighted with a color code). Tab3: SNV frequencies from the different mixtures. For the 12 mouse mtDNA mixture ratios tested, the frequencies measured for each SNV (88 in total) were evaluated. Values are actually the average of the three replicate runs. The eight positions showing a systematic frequency underestimation are highlighted in blue. Tab4: Indel frequencies from the different mixtures. For the 12 mouse mtDNA mixture ratios tested, the frequencies measured for the two indels were evaluated (positions 5’204 and 9’820). Data obtained from the triplicate, and corresponding averages and standard deviations are shown. (XLSX 61996 kb)

Additional file 8: MitoRS output file for the analysis of the CEPH family 1463. Tab1: Raw MitoRS output for the CEPH family 1463 analysis. Column headers are detailed in the Additional files 14: Table S1 legend. Data from the 17 samples are populated in consecutive columns. As explained in the Additional file 2, the rCRS position 3’107-N is deleted from the reference for proper alignment. Positions are shifted accordingly and match the rCRS numbering. Tab2: List of all variants identified in the CEPH family 1463. The parameters used to identify a variant are presented in the main text. Homoplasmic variants passing filters are highlighted in red, high frequency heteroplasmy in orange, low frequency heteroplasmy in yellow, and positions not passing filters are left in blank. For each variant, the relative coverage (in percentage), the nature of the variant (SNV or Indel), and the VarScan *p*-value are shown. The positions highlighted in blue were validated by Sanger sequencing. Tab 3 to Tab 7: Variant sharing within the CEPH family 1463. Same data as Tab2 but from selected individuals to highlight how variant are shared within the CEPH 1463 family. The data are similar to Tables 1 and 2 with extra details. Tab8: Reminder of the CEPH family 1463 pedigree. Figure 5 data are shown as a reminder of the CEPH family 1463 pedigree. Tab9: CEPH family 1463 haplogroup. Haplofind output obtained from the CEPH family 1463 fastA file generated by MitoRS. See the Additional file 10: Table S2 legend for details. (XLSX 29419 kb)

Additional file 9: MitoRS output file for the analysis of the CEPH family 884. Legend similar to the Additional file 8 but for the CEPH family 884. (XLSX 28519 kb)

Additional file 10: Table S2. Haplogroup determination for the CEPH family 1463. The fastA file generated for each individual of the CEPH family 1463 was submitted to the Haplofind tool. Note that N are considered as deletion. When the completion status is “No”, Haplofind was not able to determine the exact subhaplogroup. (DOCX 39 kb)

Additional file 11: Sanger sequencing validation for the inheritance of high level heteroplasmy SNV. Slide 1. CEPH family 1463 pedigree chart. Slide 2. Level of heteroplasmy calculated from the MitoRS data, and the Sanger sequencing data in both forward and reverse orientation. MitoRS calculated frequencies are highlighted in red (homoplasmy, > 98%) or orange (high frequency heteroplasmy, between 10% and 98%). The corresponding Sanger frequencies are highlighted in green for easier visualization. Slides 3 to 10. Chromatograms highlighting a difference between the two family members. The positions considered are shown with a black arrowhead. Slide 10. Virtual gel visualization (on a LabChip GX - Perkin Elmer) of the PCR amplification products analyzed by Sanger sequencing. (PPTX 513 kb)

Additional file 12: Table S3. Primer sequences used for qPCR. (DOCX 37 kb)

Additional file 13: MitoRS output file for the two plasmids sequenced for the benchmark of RCA. Tab1: Raw MitoRS output for plasmid1 (P1). Sample names are prefixed “Crude” when no amplification was done and “RCA” when rolling circle amplification was performed. The four replicates are named A, B, C and D. The data for the eight sequencing experiments are populated in consecutive columns. Column headers are detailed in the Additional files 14: Table S1 legend. Tab2: Raw MitoRS output for plasmid2 (P2). Same as for Plasmid1. (XLSX 6141 kb)

Additional file 14: Table S1. Structure of the output csv table. Example of an output file generate by the analysis pipeline. The table is populated for all positions of the reference genome. Chrom: Reference genome used, Position: Position in the reference genome, Covmp: Absolute depth of coverage, PercentCov: relative depth of coverage expressed as a percentage of the average coverage obtained for the sample, FilterCov: Flagging for insufficient relative coverage, Ref: Nucleotide from the reference genome, Var: Alternative nucleotide identified by VarScan, Cons: Consensus nucleotide kept by VarScan, FastA: Nucleotide kept in the exported fastA file, QDepth: Absolute depth of coverage, Reads1: Reference nucleotide coverage by mpileup, Reads2: Alternative nucleotide coverage by mpileup, Freq: Variant frequency, *P*-value: VarScan *p*-value, StrandFilter: VarScan strand filter, R1+: Reference nucleotide coverage from the positive strand, R1-: Reference nucleotide coverage from the negative strand, R2+: Alternative nucleotide coverage from the positive strand, R2-: Alternative nucleotide coverage from the negative strand. When several samples are analyzed together, each sample data are populated in consecutive columns of a single table. Full output tables from data presented in this manuscript can be found in the Additional files 7, 8, 9 and 13. (DOCX 46 kb)

Additional file 15: Figure S6. The large number of homoplasmic variants identified in the NMRI strain does not have a major impact on the mpileup reported coverage. The relative coverage reported by mpileup was plotted against each single position of the mouse reference genome for both the B6D2F1 and the NMRI datasets. The only noticeable differences are two NMRI specific “extreme” drops of coverage (positions 5’205 and 9’821) resulting from near homoplasmic indels (see the Additional file 2 for details on “extreme” coverage drops). (PPTX 264 kb)

Additional file 16: Figure S7. Coverage drop at the position 310 human C-stretch. The human sample #12878 from the CEPH family 1463 was sequenced either following the pipeline described in this paper or from a whole genome PCR free library (generated in our lab). The relative coverage reported by mpileup was plotted against each single position of the reference genome with a zoom in the C-stretch at position 310. (PPTX 233 kb)

## Abbreviations

BWA: Burrows-Wheeler Aligner
CEPH: Centre d’Etude du Polymorphisme Humain
Indel: Insertion or deletion variant
MitoRS: Mitochondrial DNA analysis based on Rolling circle amplification and Sequencing)
mtDNA: Mitochondrial DNA
NGS: Next generation sequencing
NIGMS: National Institute of General Medical Sciences
NTC: Non-template control
nucDNA: Nuclear DNA
NUMTS: Nuclear MiTochondrial DNAs
RCA: Rolling circle amplification
SNV: Single nucleotide variant
WGS: Whole genome sequencing
