# Supplementary figures and images for: MitoRS, a method for high throughput, sensitive, and accurate detection of mitochondrial DNA heteroplasmy
Source: BMC Genomics. 2017 Apr 26;18:326. doi: 10.1186/s12864-017-3695-5 (PMC5405551; doi:10.1186/s12864-017-3695-5)

## Slide 1
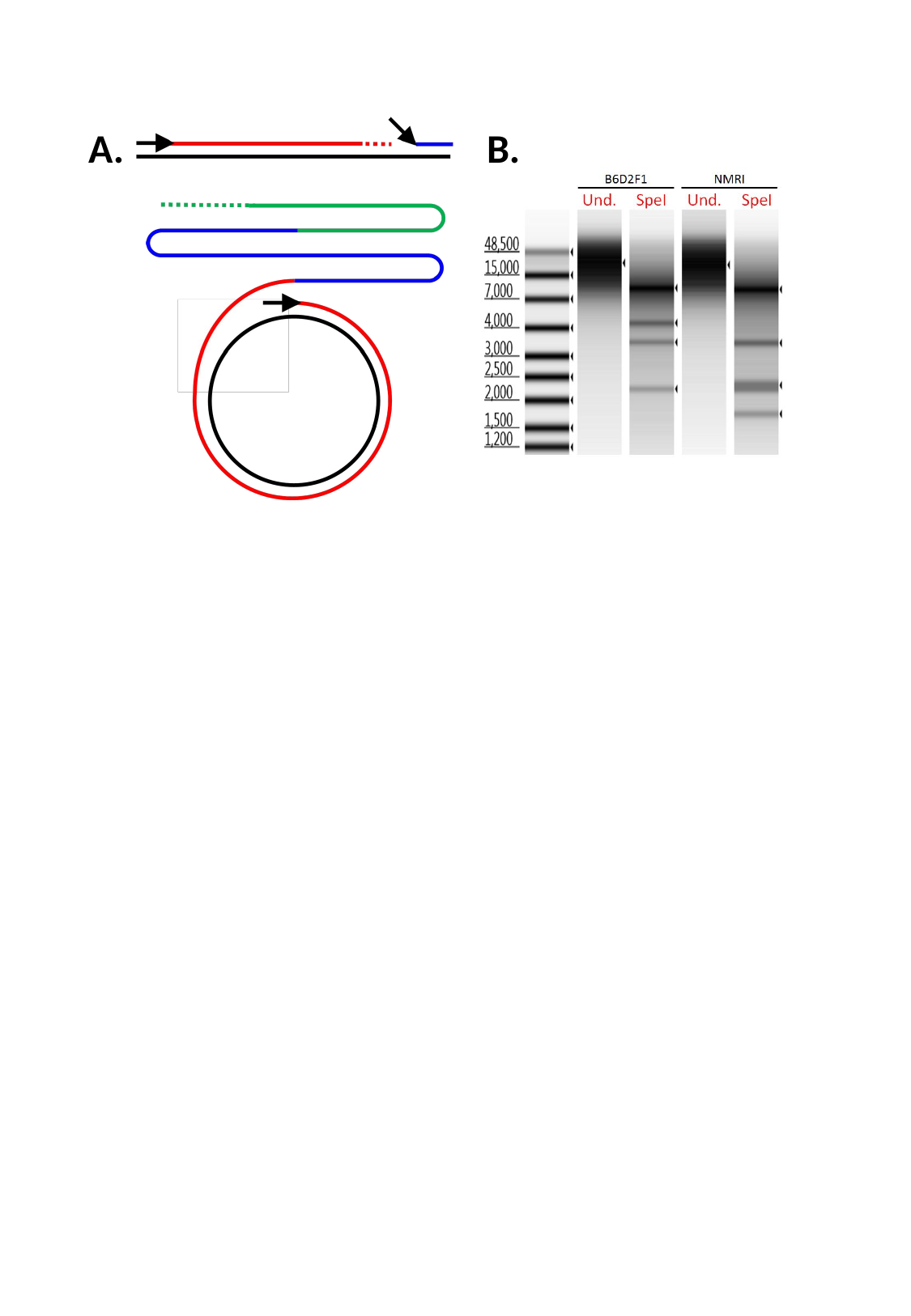

A.
B.

Supplement: Supplementary file 1 — RCA enriches circular versus linear templates. A. Principle of circular DNA enrichment. A single priming event will generate several concatenated copies of a circular template. At the opposite, a single copy will be amplified if the template DNA is linear. B. The RCA amplified material is mostly mtDNA. Digestion of the mouse DNA RCA product with the SpeI restriction endonuclease results in the expected mtDNA digestion product with only low amount of undigested DNA left. SpeI restriction digest is expected to result in four fragments for the B6D2F1 strain (7’398, 3’759, 3’105, and 2’035 bp) and five fragments for the NMRI strain (7’398, 3’105, 2’150, 2’037, and 1609 bp). Ladder sizes imprecision is in accordance with the Agilent Genomic DNA ScreenTape specifications. (PPTX 133 kb) [file 12864_2017_3695_MOESM1_ESM.pptx]

## Slide 1
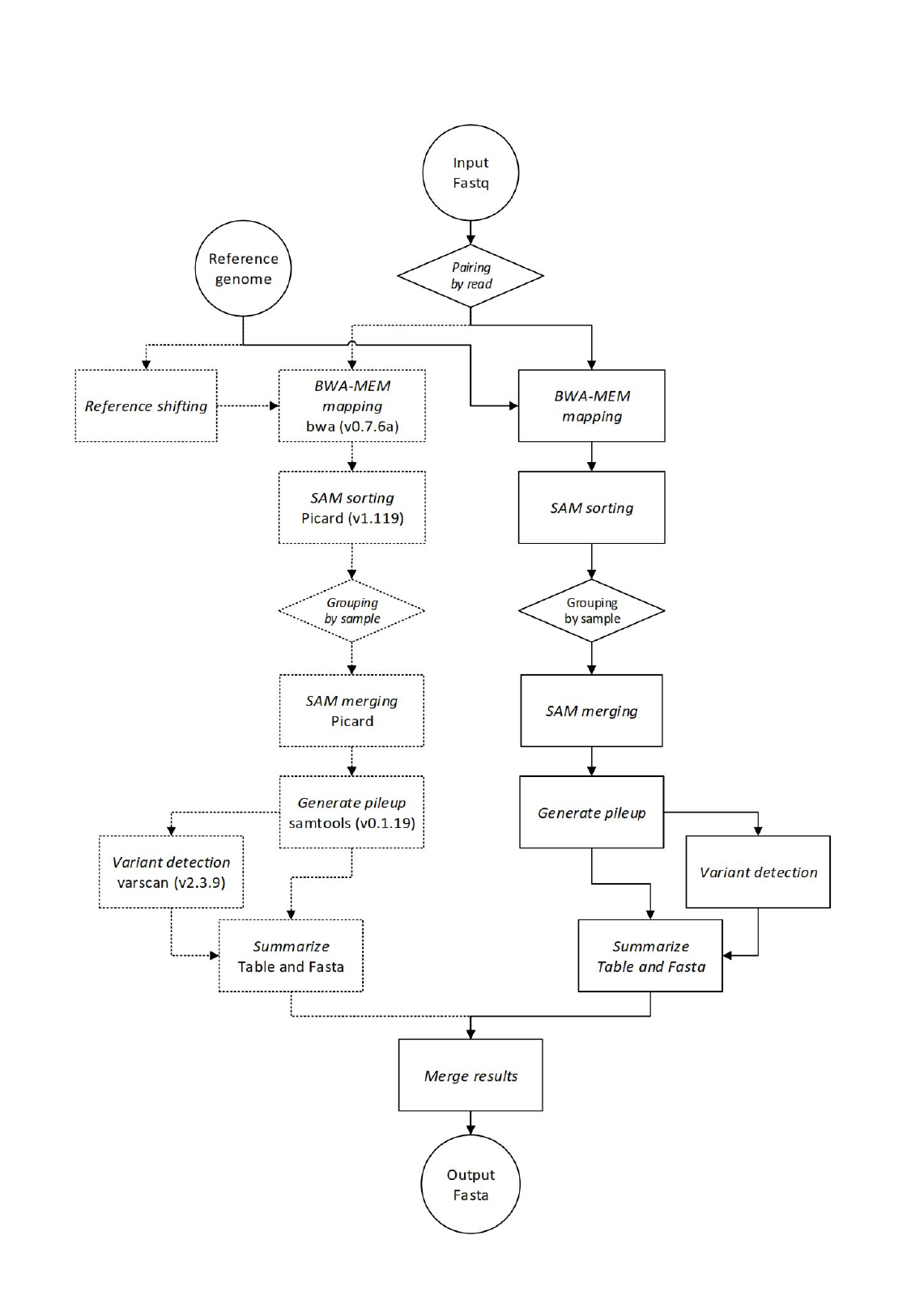

Supplement: Supplementary file 4 — Overview of the MitoRS analysis methods. FastQ files are aligned with BWA to the original, and an origin-shifted version, of the DNA reference sequence (refer to Additional file 2 for details). Variant frequency is evaluated by samtools mpileup. Low frequency variants are finally identified using VarScan 2. Datasets generated from both the original reference and the shifted reference are merged, keeping only the per position values from the dataset with the highest coverage. Data are further processed to generate 1) a csv file summarizing the sequencing results observed at each individual mitochondrial DNA position and 2) a fastA file representing the corresponding consensus sequence. More details can be found in the Methods section. (PPTX 264 kb) [file 12864_2017_3695_MOESM4_ESM.pptx]

## Slide 1
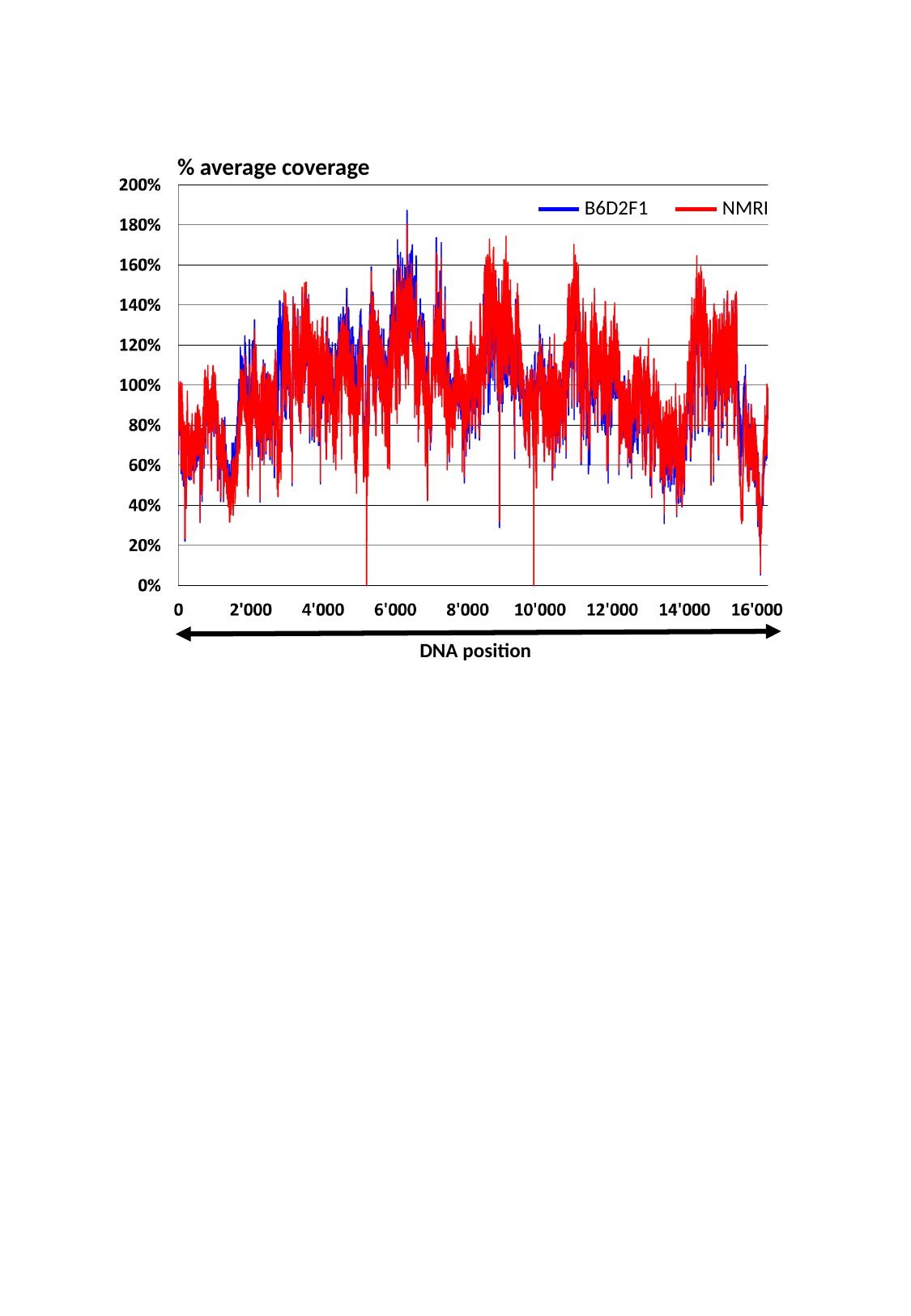

% average coverage
B6D2F1
NMRI
DNA position

Supplement: Supplementary file 15 — The large number of homoplasmic variants identified in the NMRI strain does not have a major impact on the mpileup reported coverage. The relative coverage reported by mpileup was plotted against each single position of the mouse reference genome for both the B6D2F1 and the NMRI datasets. The only noticeable differences are two NMRI specific “extreme” drops of coverage (positions 5’205 and 9’821) resulting from near homoplasmic indels (see the Additional file 2 for details on “extreme” coverage drops). (PPTX 264 kb) [file 12864_2017_3695_MOESM15_ESM.pptx]

## Slide 1
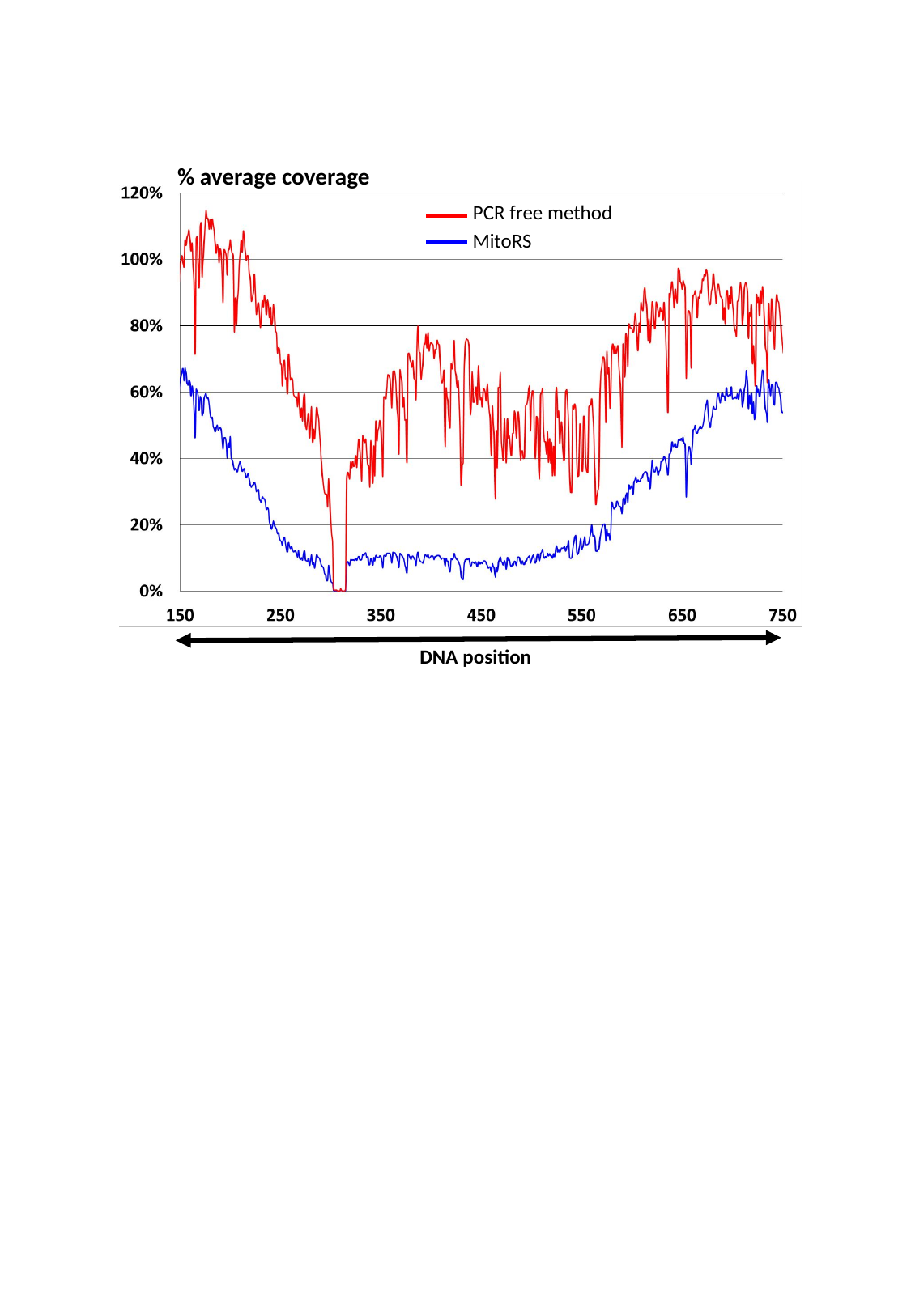

% average coverage
PCR free method
MitoRS
DNA position

Supplement: Supplementary file 16 — Coverage drop at the position 310 human C-stretch. The human sample #12878 from the CEPH family 1463 was sequenced either following the pipeline described in this paper or from a whole genome PCR free library (generated in our lab). The relative coverage reported by mpileup was plotted against each single position of the reference genome with a zoom in the C-stretch at position 310. (PPTX 233 kb) [file 12864_2017_3695_MOESM16_ESM.pptx]
