# Supplementary material for: MitoRS, a method for high throughput, sensitive, and accurate detection of mitochondrial DNA heteroplasmy
Source: BMC Genomics. 2017 Apr 26;18:326. doi: 10.1186/s12864-017-3695-5 (PMC5405551; doi:10.1186/s12864-017-3695-5)
Supplement: Supplementary file 2 — Supporting Information. (DOCX 56 kb) [file 12864_2017_3695_MOESM2_ESM.docx]

# Additional file 2: Supporting Information

## MitoRS method overview

For the amplification of mtDNA, the RCA reaction is run from 5 ng total DNA. This step is very simple to setup and typically performed with several tens of samples in a 96 well plate format. A single reaction well per sample is necessary to amplify the whole mtDNA. Note that we did formally evaluate whether RCA priming was universal. The kit we used contains human mitochondrial DNA-specific primers but we evaluated and confirmed that it enables the amplification of mouse mitochondrial DNA and even plasmid DNA with the same efficiency and high yield. Tests performed using (phosphorothioate protected) random hexamers instead of the human primer did not further improve that yield (data not shown).

The amplified mitochondrial DNA is processed into sequencing libraries using the Nextera XT kit from Illumina. This tagmentation-based procedure is in line with a high-throughput strategy: it requires less than a day of work in the laboratory including quality control of the libraries, it is performed in a 96 well plate format, and it allows the multiplexing of more than 96 samples at once. We chose Nextera XT given its recognized robustness, ease of use and high throughput. However, any other library generation strategy could be deployed. Samples are pooled and sequenced on a HiSeq 2500 paired end run of 2 x 150 cycles. The equivalent of ~120 samples are loaded per flow cell lane.

Following demultiplexing, the resulting fastQ files are aligned to the reference sequence with the Burrows-Wheeler Aligner(BWA [[1](#_ENREF_2_1)], version 0.7.4) using default parameters. No nucDNA mapping filter is applied since the relative coverage of both genomes, largely in favor of mtDNA, is sufficient to prevent reads of NUMTs origin from interfering with mtDNA variant detection (see below). Due to the small size of the mitochondrial genome (~ 16.5 kb) and the high coverage obtained, many duplicate reads are detected and kept for the analysis. The resulting bam files are filtered by mpileup for high quality bases and alignment.

Variants are called with VarScan 2 (version 2.3.6) using default parameters (including strand filtering). The choice of the variant caller is key for the variant analysis accuracy. Heteroplasmic mtDNA variants are somehow similar to somatic variants (as found in cancer biopsies for instance) in the fact that their frequencies can vary extensively and be extremely low (as opposed to nuclear DNA variants which frequency must be either 0%, 50% or 100%), and that sequencing datasets are generally very deep. In the context of somatic variant analysis, it has been demonstrated that VarScan 2 is amongst the best performing algorithm, being able to accurately identify variants at frequency as low as 1% [[2](#_ENREF_2_2)].

In addition, we included relative coverage lower thresholds to flag or exclude the few low complexity regions (as for instance the position 309 C-stretch from human mtDNA) which are less covered than the majority of the mitochondrial genome. The aim is to prevent false positive variant calling resulting from improper sequence alignment in low complexity regions. This coverage threshold strategy is further detailed below.

Finally, special attention was given to the final output csv file designed to be handled without advanced bioinformatics tools. Each single position from the reference genome is reported with its coverage (and the corresponding flagging described below), variant frequency and several other VarScan outputs. All the samples from a given study are concatenated into the same table, facilitating their comparison. Sorting and filtering tools from any spreadsheet management software are sufficient even for advanced data analysis. The structure of the output data can be found in the attached Additional Files 7, 8, 9 and 13, and as an example in the Additional file 14: Table S1. In addition, a per sample consensus fastA file is produced which can be used for haplotype calling or simple sequence comparison. Importantly, these data reporting steps do not require any manual curation and can be directly used for further analysis.

A complete summary of the laboratory procedures and the analysis pipeline developed for MitoRS can be found in the Supporting Information section and in the Additional file 3: Figures S1 and Additional file 4: Figure S2.

## Analysis parameters

### Mitochondrial DNA circular reference

Mitochondrial DNA being circular, sequencing reads cannot be mapped properly when landing too close by the origin, which is positioned at the extremities of the linearized reference sequence. When approaching the origin, the coverage drops accordingly. To solve this problem, the analysis pipeline was systematically run against two linear references: the original one, and a second one with the origin shifted to the center of the linear reference (Additional file 4: Figure S2). We confirmed that the two datasets are strictly similar for the central regions where the coverage is the same i.e. regions where the exact same reads can be taken into consideration. Results from the two references are subsequently merged, keeping only the data coming from the dataset (original or shifted reference) in which the mpileup coverage is the highest.

### Base alignment quality option by mpileup

Several 1-3 bp extreme drops of coverage (coverage = 0) were observed in some datasets (for instance, see NMRI mtDNA on Additional file 15: Figure S6). These are occurring at the position following an indel when the next nucleotide is identical to the nucleotide involved in the indel. This situation results in a BWA mapping uncertainty flagged by mpileup which subsequently excludes the corresponding positions from the analysis. Turning off the computation of base alignment quality by mpileup (BAQ, -B option) does restore the coverage of these missed nucleotides (data not shown). However, it does also affect the accuracy of variant detection by introducing some false positive in regions where indel alignment is complex. We therefore decided not to apply this option.

### Low complexity mtDNA regions

The coverage plots generated from human samples did reveal a 5-10 bp wide extreme drops of coverage (less than 0.5% of the average coverage) around a poly-C region centered at position 310 (Additional file 16: Figure S7). This phenomenon is observed for all human samples we have tested so far, though its extent depends on the exact C-stretch sequence of the individual mtDNA analyzed. The human position 310 mtDNA C-stretch is known to be problematic for NGS analysis (see Goto et al. for instance [[3](#_ENREF_2_3)]). It originates from the difficulties of mapping sequencing reads specifically at this position, whereas it does not affect to a similar extent other C-stretches such as positions 961, 3’572 or 16’189. The source of this phenomenon cannot be imputed to RCA or library PCR amplification as it also observed with sequencing libraries generated with a PCR free method, though the surrounding global lower coverage is less prominent (Additional file 16: Figure S7).

These dramatic drops of coverage can result in short genomic regions which even performant variant callers cannot analyze correctly. Varscan does indeed only make a call when the read depth at a position is more than 8x (default parameter). It will then report any variant found, without penalizing the mapping uncertainties indicated by the fact that the coverage is abnormally low compared to the surrounding region. We therefore decided to include a minimal relative coverage filtering threshold in order to prevent false positive variant calling in such low complexity regions with too low coverage. Practically, the coverage of each position is computed as the percentage of the average coverage observed over the whole reference genome for a given sample. A warning threshold is set at 10% (≈ 30 to 300X coverage) and an exclusion threshold at 1%. The impact on human mtDNA analysis is actually moderate with less than 1.5% of the genome being flagged (≈ 200 to 250 positions for the 16’568 bp genome, most of them corresponding to the region centered around the 309 C-stretch) and less than 0.15% excluded (≈ 10 to 15 positions). Accordingly, all position are marked with a “GOOD”, “WARNING” or “EXCLUDE” comment in the output csv file.

### Human mtDNA reference

The human reference sequence used was the rCRS sequence (NC_012920.1) in which the N at position 3’107 was removed because it interferes with proper mpileup data analysis. The computation of base alignment quality (see above) would otherwise result in four adjacent positions with a null coverage, preventing any variant detection around that position. We do not intend to propose here the revision the human mitochondrial reference sequence [[4](#_ENREF_2_4), [5](#_ENREF_2_5)] but simply remove a practical constraint. It has no impact for haplotype calling since it is not a vcf-type file but the output fastA file which is used to call for haplotype (the 3’107-N deletion is then simply handled as a deletion). Note that all positions reported here are corrected to match the rCRS numbering.

## Contaminating reads originating from RCA

### Unspecific amplification

In the course of the pipeline benchmarking using plasmid DNA, we repeatedly observed an amplification product in the non-template control (NTC). This amplification product was used to generate a library which was subsequently sequenced. The resulting reads could clearly be mapped on plasmid DNA but only very partially to the 2 plasmids we actually assayed in this test (see below). Such amplification in the NTC is not always observed but correlates with REPLI-g Mitochondrial DNA kit lot used. We aligned the read to several expression plasmid reference sequences and obtained very good matches over large regions, though we did not intend to identify which exact plasmid these contaminating reads were corresponding to. This makes us confident that this signal is generated from the Phi29 expression plasmid DNA co-purified with the enzyme. Such Phi29 polymerase contamination has been previously described [[6](#_ENREF_2_6)].

### Consequences on RCA accuracy analysis

While comparing the differences in variant frequency between the crude and the RCA treated DNA, we observed two non-concordant positions with a frequency difference up to 24% (marked with a star on Figure 3A). In both cases, it lies within the plasmid origin of replication and corresponds to an “A” to “G” transition. The same region can also be mapped from the reads generated from the NTC condition for which we found a “G” at near homoplasmy (Additional file 5: Figure S4B). From these observations and the alignment of the reference sequences, we could conclude that the two plasmids have an “A” at this position. This hypothesis could be confirmed from the sequencing data generated from the same samples but without the RCA step. The reads with a “G” are therefore not an RCA error, but actually contaminating reads originating from the Phi29 polymerase enzyme preparation. Note that the variant frequency difference between the two plasmids is the effect of the proportion of mapped genuine reads versus contaminating reads, i.e. to the level of NTC contamination.

This contamination is not comfortable to work with because it is complex to identify any possible similarity between the negative control reads and the sequence of interest. However, by mapping the NTC reads against the human and mouse mtDNA reference sequences, only very few matches were found, 0.02% for mouse mtDNA and 0.002% for human DNA. This confirms that the NTC contamination does not interfere with accurate mtDNA analysis.

## Variant detection in unmixed mouse mtDNA preparation

### Unmixed mouse mtDNA sample analysis

DNA samples for each B6D2F1 and NMRI strains were sequenced in order to establish a comparison with the mouse mtDNA reference sequence (accession NC_005089). The average coverage was around 3’000X. When considering SNV at a frequency > 0.5% and a VarSan p-value < 0.001 (see plasmid DNA analysis), the B6D2F1 sample differs only at a single position compared to the mouse mtDNA reference sequence. This SNV is heteroplasmic with a frequency ~ 50%. We could also identify six low frequency indels (maximum frequency ~ 7%) when applying the 2% frequency threshold established by plasmid DNA sequencing. The NMRI sample is very different with 89 SNV, all being homoplasmic (frequency > 99.5%) but one with low frequency heteroplasmy (frequency ~ 1%). There are also eight indels, all heteroplasmic though two of them are high frequencies (~ 84% and ~ 98%). These results were obtained from three independent RCA replicates and are available in the Additional file 7. Note that the sequencing coverage pattern was very similar between both strains despite the high divergence between both samples (Additional file 15: Figure S6).

Interestingly, the five low frequency indels found in the B6D2F1 strain are also found at a very similar frequency in the NMRI strain (see the Additional file 7). This frequency similarity is unexpected given the large divergence between the two mtDNA genomes. Given that all five are insertions directly adjacent to a large homopolymeric stretch, they could actually be artifacts, either from the lab procedures and/or from improper variant detection. They are here considered as false positives. For instance, the position 5’171 insertion, a single A insertion in front of a stretch of 11 A, occurs at ~ 7% in both mouse strains. This suggests that the indel detection lower threshold at 2% could result in inaccurate variant calls. It is challenging to benchmark a threshold since such type of indel bias is highly sequence specific and it will undoubtedly be influenced by the homopolymeric stretch length and context.

The only low frequency SNV identified is the position 6’158 in NMRI (frequency = 1.2%). It is not at all detected in the B6D2F1 mtDNA. Its frequency nicely follow the mixture ratios until the 50% ratio after which it drops below the lower limit of detection (data not shown). This strongly suggests that this is a genuine variant and that no false positive SNV are found in this mouse dataset.

### Frequency ratio recalibration

Knowing the exact ratio of both mtDNA in each mixture is key for evaluating the accuracy of the pipeline. The initial estimation was performed from qPCR data and revealed that the mtDNA concentrations were very similar in the total DNA extracted for both strains (Figure 1A).

However, quantitative PCR is not accurate enough to capture small differences between samples. We therefore recalibrated the input ratios by correcting for the actual B6D2F1 and NMRI mtDNA concentrations. This correction was based on the 50% ratio since it is a priori the most precise dilution. The expected 50% ratio actually resulted in a median frequency of 44.4% when considering all the 88 SNV between B6D2F1 and NMRI. This means that the NMRI mtDNA concentration was overestimated 1.25-fold compared to the B6D2F1 mtDNA concentration. The corresponding correction factor was then applied to all 12 ratio mixtures presented in Figure 4.

## References

1. Li H: **Aligning sequence reads, clone sequences and assembly contigs with BWA-MEM**. *[q-bioGN]* 2013, **arXiv:1303.3997**.

2. Stead LF, Sutton KM, Taylor GR, Quirke P, Rabbitts P: **Accurately identifying low-allelic fraction variants in single samples with next-generation sequencing: applications in tumor subclone resolution**. *Human mutation* 2013, **34**(10):1432-1438.

3. Goto H, Dickins B, Afgan E, Paul IM, Taylor J, Makova KD, Nekrutenko A: **Dynamics of mitochondrial heteroplasmy in three families investigated via a repeatable re-sequencing study**. *Genome biology* 2011, **12**(6):R59.

4. Bandelt HJ, Kloss-Brandstatter A, Richards MB, Yao YG, Logan I: **The case for the continuing use of the revised Cambridge Reference Sequence (rCRS) and the standardization of notation in human mitochondrial DNA studies**. *Journal of human genetics* 2014, **59**(2):66-77.

5. Behar DM, van Oven M, Rosset S, Metspalu M, Loogvali EL, Silva NM, Kivisild T, Torroni A, Villems R: **A "Copernican" reassessment of the human mitochondrial DNA tree from its root**. *American journal of human genetics* 2012, **90**(4):675-684.

6. Takahashi H, Yamazaki H, Akanuma S, Kanahara H, Saito T, Chimuro T, Kobayashi T, Ohtani T, Yamamoto K, Sugiyama S *et al*: **Preparation of Phi29 DNA polymerase free of amplifiable DNA using ethidium monoazide, an ultraviolet-free light-emitting diode lamp and trehalose**. *PloS one* 2014, **9**(2):e82624.
