# Supplementary material for: MitoRS, a method for high throughput, sensitive, and accurate detection of mitochondrial DNA heteroplasmy
Source: BMC Genomics. 2017 Apr 26;18:326. doi: 10.1186/s12864-017-3695-5 (PMC5405551; doi:10.1186/s12864-017-3695-5)
Supplement: Supplementary file 3 — Overview of the MitoRS wet lab method. Total DNA (5 ng) is amplified by RCA. Sequencing libraries are subsequently generated using the Nextera XT kit from Illumina, starting from 1 ng amplification product. Libraries are pooled at equimolar ratios and sequenced on a HiSeq 2500 (Illumina) using rapid mode for a paired-end run of 2 x 150 cycles. (PPTX 40 kb) [file 12864_2017_3695_MOESM3_ESM.pptx]

## Slide 1
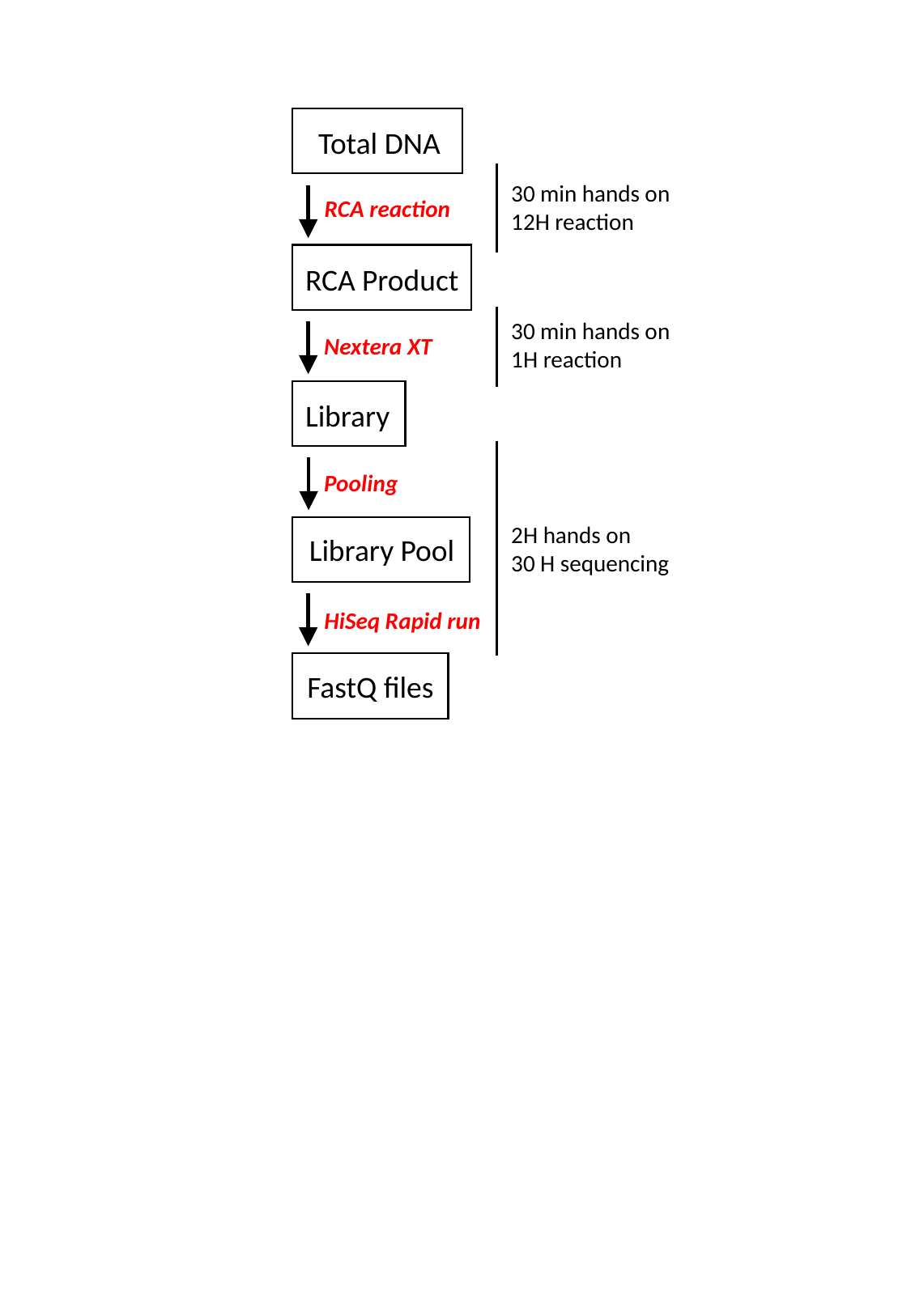

Total DNA
30 min hands on
12H reaction
RCA reaction
RCA Product
30 min hands on
1H reaction
Nextera XT
Library
Pooling
2H hands on
30 H sequencing
Library Pool
HiSeq Rapid run
FastQ files
