# Supplementary material for: MitoRS, a method for high throughput, sensitive, and accurate detection of mitochondrial DNA heteroplasmy
Source: BMC Genomics. 2017 Apr 26;18:326. doi: 10.1186/s12864-017-3695-5 (PMC5405551; doi:10.1186/s12864-017-3695-5)
Supplement: Supplementary file 5 — The RCA procedure is robust. A. RCA does not introduce technical variability. Each individual plasmid DNA was run as four independent replicates for both conditions (with or without RCA). The technical reproducibility of the variant frequency call was evaluated by calculating the standard deviation within the four replicates. Value are plotted for each individual position of the reference sequence, for the two plasmids sequenced. Data are directly extracted from mpileup analysis applying a VarScan p-value threshold of 0.001. Left panels: plasmid 1, right panels: plasmid 2, top panels: crude plasmid DNA (no RCA amplification), bottom panels: RCA amplified plasmid DNA. B. Origin of the nonspecific RCA products. The sequences surrounding the unique position for which a large difference was observed between the crude plasmid DNAs and the RCA products were aligned. The sequence obtained from contaminating NTC reads is shown. The common (between the two plasmids) non-accurate position is boxed (“G” in the NTC, “A” in the two plasmids). This region is part of the plasmid DNA origin of replication. (PPTX 178 kb) [file 12864_2017_3695_MOESM5_ESM.pptx]

## Slide 1
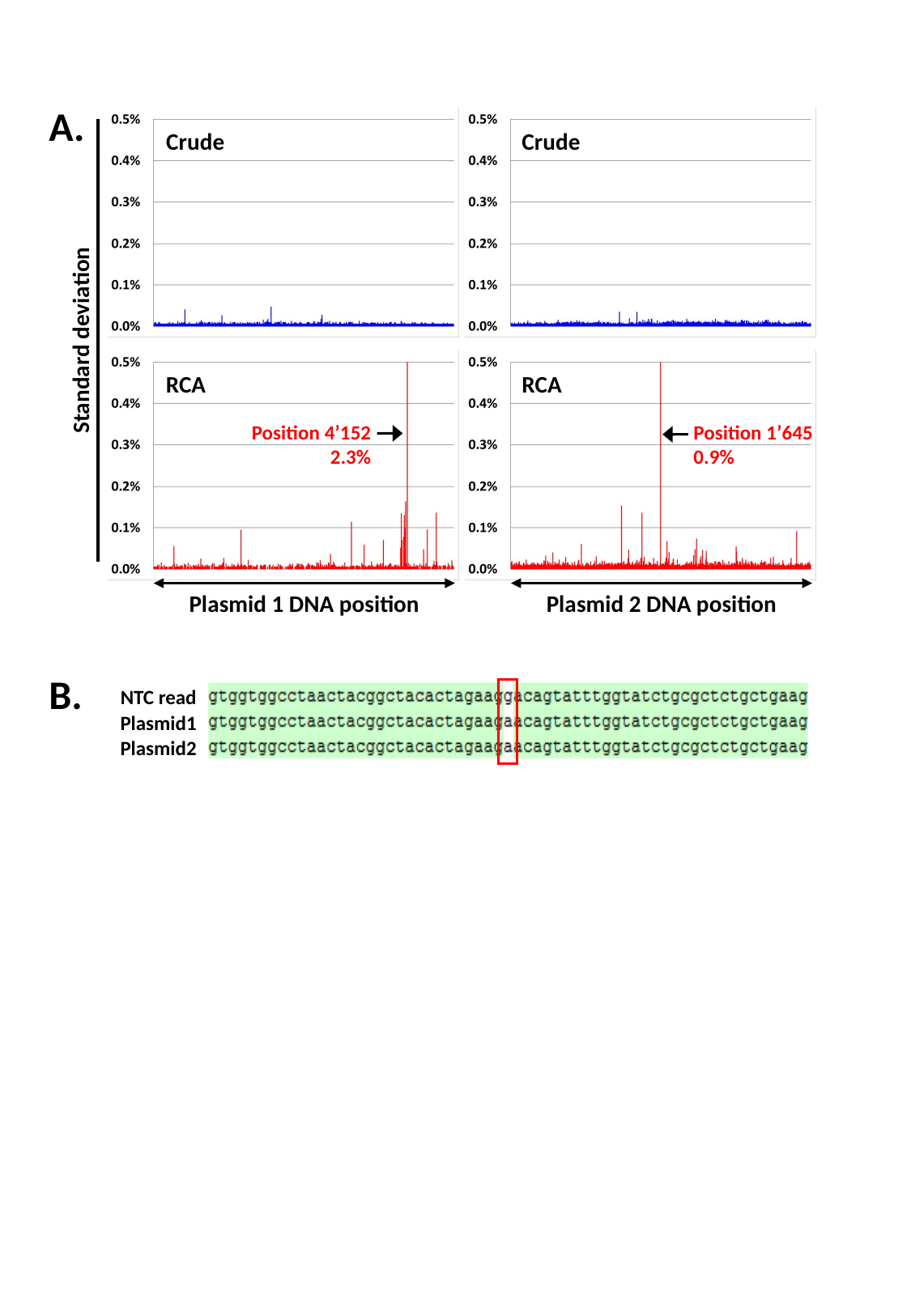

A.
Crude
Crude
Standard deviation
RCA
RCA
Position 4’152
2.3%
Position 1’645
0.9%
Plasmid 1 DNA position
Plasmid 2 DNA position
B.
NTC read
Plasmid1
Plasmid2
