# Supplementary material for: MitoRS, a method for high throughput, sensitive, and accurate detection of mitochondrial DNA heteroplasmy
Source: BMC Genomics. 2017 Apr 26;18:326. doi: 10.1186/s12864-017-3695-5 (PMC5405551; doi:10.1186/s12864-017-3695-5)
Supplement: Supplementary file 6 — Mouse SNV frequencies are homogenous within the 88 position analyzed. A. Individual SNV frequency. For the 12 mouse mtDNA mixture ratios tested, the frequencies measured for each SNV (88 in total) were plotted and summarized as a boxplot. The boxplot whiskers highlight the extreme values (min and max). Note that the scale is different for each plot. The eight positions showing the highest frequency underestimation are highlighted in blue. These data were used to build the Fig. 4. The raw data are available from the Additional file 7. B. The eight SNV with underestimated frequency are located into two dense clusters. For each 88 SNV, the deviation from the theoretical frequency was calculated and plotted versus their position in the mitochondrial genome. The eight positions showing a systematic frequency underestimation highlighted in A. are also shown in blue. They cluster into two very short genomic regions. (PPTX 484 kb) [file 12864_2017_3695_MOESM6_ESM.pptx]

## Slide 1
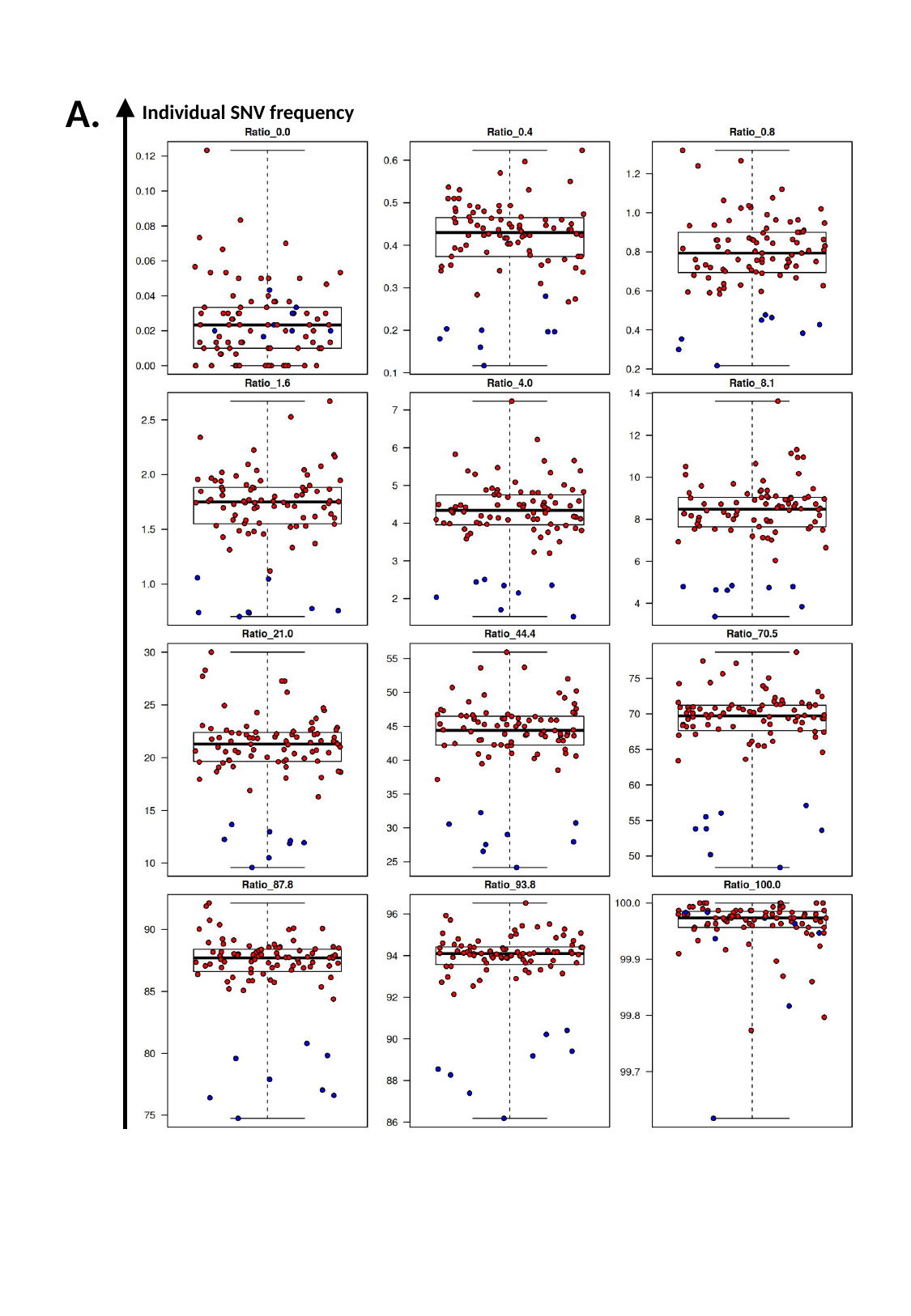

A.
Individual SNV frequency

## Slide 2
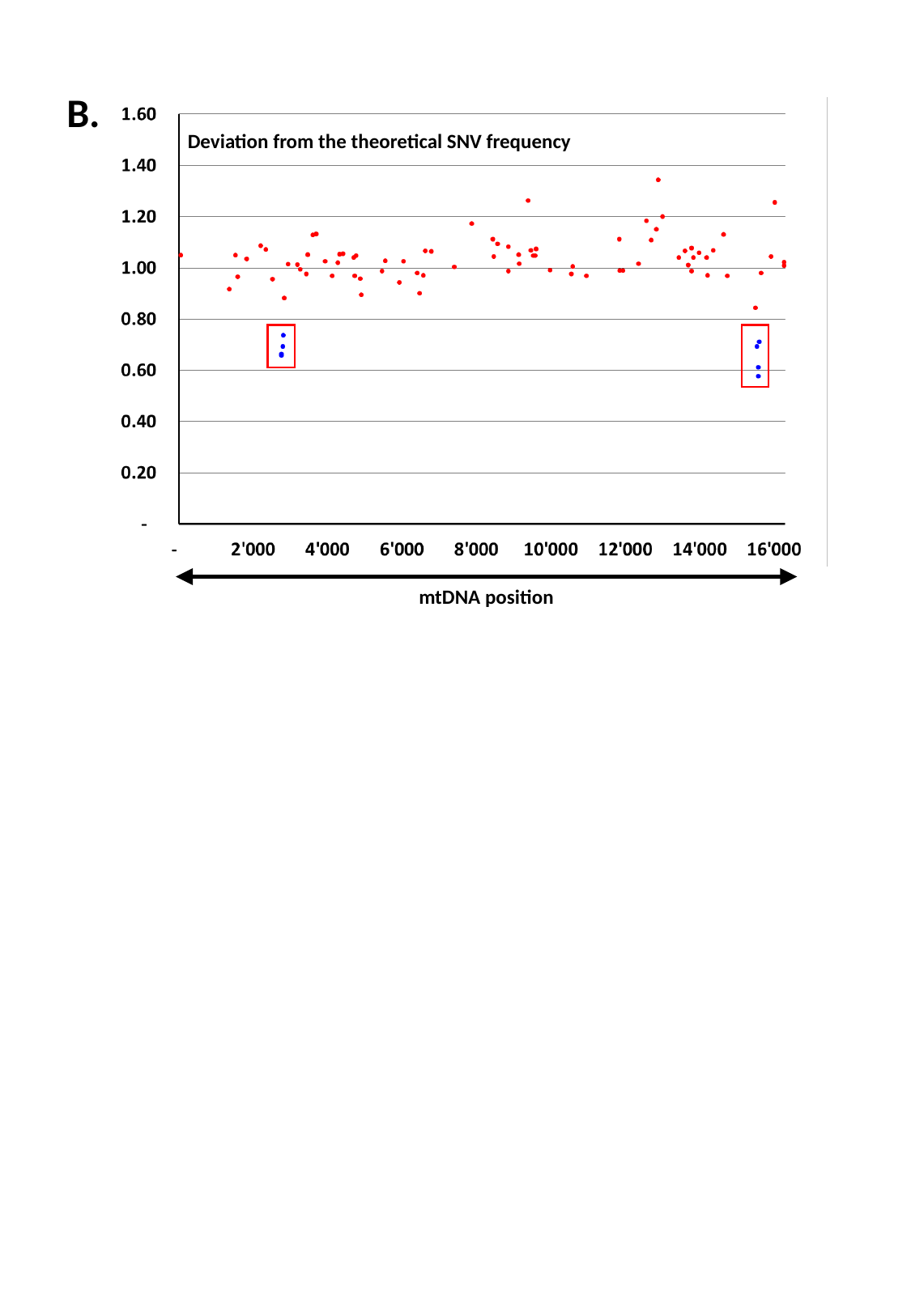

B.
Deviation from the theoretical SNV frequency
mtDNA position
