# Supplementary material for: MitoRS, a method for high throughput, sensitive, and accurate detection of mitochondrial DNA heteroplasmy
Source: BMC Genomics. 2017 Apr 26;18:326. doi: 10.1186/s12864-017-3695-5 (PMC5405551; doi:10.1186/s12864-017-3695-5)
Supplement: Supplementary file 10 — Haplogroup determination for the CEPH family 1463. The fastA file generated for each individual of the CEPH family 1463 was submitted to the Haplofind tool. Note that N are considered as deletion. When the completion status is “No”, Haplofind was not able to determine the exact subhaplogroup. (DOCX 39 kb) [file 12864_2017_3695_MOESM10_ESM.docx]

## Table S2

**Haplogroup determination for the CEPH family 1463**

| Identifier | Haplogroup | Score | Mutations | Complete |
| --- | --- | --- | --- | --- |
| GM12877 | H1ap1 | 0.7 | 73A; 146T; 195T; 247G; 296_312del; 769G; 825T; 1018G; 2706A; 2758G; 2885T; 2989A; 3010A; 3144G; 3594C; 4104A; 4312C; 7028C; 7146A; 7256C; 7521G; 8468C; 8655C; 8701A; 9540T; 10398A; 10664C; 10688G; 10810T; 10873T; 10915T; 11719G; 11914G; 12468C; 12705C; 13105A; 13276A; 13506C; 13650C; 14766C; 15497A; 16129G; 16187C; 16223C; 16230A; 16278C; 16311T | No |
| GM12878 | H13a1a1a | 1 | 73A; 146T; 195T; 247G; 303_315del; 769G; 825T; 1018G; 2259T; 2706A; 2758G; 2885T; 3594C; 4104A; 4312C; 4745G; 7028C; 7146A; 7256C; 7337A; 7521G; 8468C; 8655C; 8701A; 9540T; 10398A; 10664C; 10688G; 10810T; 10873T; 10915T; 11719G; 11914G; 12705C; 13105A; 13276A; 13326C; 13506C; 13650C; 13680T; 14766C; 14831A; 14872T; 16023A; 16129G; 16187C; 16189T; 16223C; 16230A; 16278C; 16311T; 16519T | No |
| GM12879 | H13a1a1a | 1 | 73A; 146T; 195T; 247G; 303_315del; 769G; 825T; 1018G; 2259T; 2706A; 2758G; 2885T; 3594C; 4104A; 4312C; 4745G; 7028C; 7146A; 7256C; 7337A; 7521G; 8468C; 8655C; 8701A; 9540T; 10398A; 10664C; 10688G; 10810T; 10873T; 10915T; 11719G; 11914G; 12705C; 13105A; 13276A; 13326C; 13506C; 13650C; 13680T; 14766C; 14831A; 14872T; 16129G; 16187C; 16189T; 16223C; 16230A; 16278C; 16311T; 16519T | No |
| GM12880 | H13a1a1a | 1 | 73A; 146T; 195T; 247G; 308_314del; 769G; 825T; 1018G; 2259T; 2706A; 2758G; 2885T; 3594C; 4104A; 4312C; 4745G; 7028C; 7146A; 7256C; 7337A; 7521G; 8468C; 8655C; 8701A; 9540T; 10398A; 10664C; 10688G; 10810T; 10873T; 10915T; 11719G; 11914G; 12705C; 13105A; 13276A; 13326C; 13506C; 13650C; 13680T; 14766C; 14831A; 14872T; 16129G; 16187C; 16189T; 16223C; 16230A; 16278C; 16311T; 16519T | No |
| GM12881 | H13a1a1a | 1 | 73A; 146T; 195T; 247G; 303_315del; 769G; 825T; 1018G; 2259T; 2706A; 2758G; 2885T; 3594C; 4104A; 4312C; 4745G; 7028C; 7146A; 7256C; 7337A; 7521G; 8468C; 8655C; 8701A; 9540T; 10398A; 10664C; 10688G; 10810T; 10873T; 10915T; 11719G; 11914G; 12705C; 13105A; 13276A; 13326C; 13506C; 13650C; 13680T; 14766C; 14831A; 14872T; 16129G; 16187C; 16189T; 16223C; 16230A; 16278C; 16311T; 16519T | No |
| GM12882 | H13a1a1a | 1 | 73A; 146T; 195T; 247G; 303_315del; 769G; 825T; 1018G; 2259T; 2706A; 2758G; 2885T; 3594C; 4104A; 4312C; 4745G; 7028C; 7146A; 7256C; 7337A; 7521G; 8468C; 8655C; 8701A; 9540T; 10398A; 10664C; 10688G; 10810T; 10873T; 10915T; 11719G; 11914G; 12705C; 13105A; 13276A; 13326C; 13506C; 13650C; 13680T; 14766C; 14831A; 14872T; 16129G; 16187C; 16189T; 16223C; 16230A; 16278C; 16311T; 16519T | No |
| GM12883 | H13a1a1a | 1 | 73A; 146T; 195T; 247G; 303_315del; 769G; 825T; 1018G; 2259T; 2706A; 2758G; 2885T; 3594C; 4104A; 4312C; 4745G; 7028C; 7146A; 7256C; 7337A; 7521G; 8468C; 8655C; 8701A; 9540T; 10398A; 10664C; 10688G; 10810T; 10873T; 10915T; 11719G; 11914G; 12705C; 13105A; 13276A; 13326C; 13506C; 13650C; 13680T; 14766C; 14831A; 14872T; 16129G; 16187C; 16189T; 16223C; 16230A; 16278C; 16311T; 16519T | No |
| GM12884 | H13a1a1a | 1 | 73A; 146T; 195T; 247G; 307_314del; 769G; 825T; 1018G; 2259T; 2706A; 2758G; 2885T; 3594C; 4104A; 4312C; 4745G; 7028C; 7146A; 7256C; 7337A; 7521G; 8468C; 8655C; 8701A; 9540T; 10398A; 10664C; 10688G; 10810T; 10873T; 10915T; 11719G; 11914G; 12705C; 13105A; 13276A; 13326C; 13506C; 13650C; 13680T; 14766C; 14831A; 14872T; 16129G; 16187C; 16189T; 16223C; 16230A; 16278C; 16311T; 16519T | No |
| GM12885 | H13a1a1a | 1 | 73A; 146T; 195T; 247G; 303_315del; 769G; 825T; 1018G; 2259T; 2706A; 2758G; 2885T; 3594C; 4104A; 4312C; 4745G; 7028C; 7146A; 7256C; 7337A; 7521G; 8468C; 8655C; 8701A; 9540T; 10398A; 10664C; 10688G; 10810T; 10873T; 10915T; 11719G; 11914G; 12705C; 13105A; 13276A; 13326C; 13506C; 13650C; 13680T; 14766C; 14831A; 14872T; 16129G; 16187C; 16189T; 16223C; 16230A; 16278C; 16311T; 16519T | No |
| GM12886 | H13a1a1a | 1 | 73A; 146T; 195T; 247G; 303_315del; 769G; 825T; 1018G; 2259T; 2706A; 2758G; 2885T; 3594C; 4104A; 4312C; 4745G; 7028C; 7146A; 7256C; 7337A; 7521G; 8468C; 8655C; 8701A; 9540T; 10398A; 10664C; 10688G; 10810T; 10873T; 10915T; 11719G; 11914G; 12705C; 13105A; 13276A; 13326C; 13506C; 13650C; 13680T; 14766C; 14831A; 14872T; 16129G; 16187C; 16189T; 16223C; 16230A; 16278C; 16311T; 16519T | No |
| GM12887 | H13a1a1a | 1 | 73A; 146T; 195T; 247G; 303_315del; 769G; 825T; 1018G; 2259T; 2706A; 2758G; 2885T; 3594C; 4104A; 4312C; 4745G; 7028C; 7146A; 7256C; 7337A; 7521G; 8468C; 8655C; 8701A; 9540T; 10398A; 10664C; 10688G; 10810T; 10873T; 10915T; 11719G; 11914G; 12705C; 13105A; 13276A; 13326C; 13506C; 13650C; 13680T; 14766C; 14831A; 14872T; 16129G; 16187C; 16189T; 16223C; 16230A; 16278C; 16311T; 16519T | No |
| GM12888 | H13a1a1a | 1 | 73A; 146T; 195T; 247G; 303_315del; 769G; 825T; 1018G; 2259T; 2706A; 2758G; 2885T; 3594C; 4104A; 4312C; 4745G; 7028C; 7146A; 7256C; 7337A; 7521G; 8468C; 8655C; 8701A; 9540T; 10398A; 10664C; 10688G; 10810T; 10873T; 10915T; 11719G; 11914G; 12705C; 13105A; 13276A; 13326C; 13506C; 13650C; 13680T; 14766C; 14831A; 14872T; 16129G; 16187C; 16189T; 16223C; 16230A; 16278C; 16311T; 16519T | No |
| GM12889 | H31a | 1 | 72G; 73A; 152T; 247G; 303_315del; 769G; 825T; 1018G; 2706A; 2758G; 2885T; 3594C; 4104A; 4312C; 7028C; 7146A; 7256C; 7521G; 7930T; 8468C; 8655C; 8701A; 9540T; 10398A; 10664C; 10688G; 10771G; 10810T; 10873T; 10915T; 11719G; 11914G; 12705C; 13105A; 13276A; 13506C; 13650C; 14766C; 16129G; 16187C; 16189T; 16223C; 16230A; 16278C; 16311T; 16319A | No |
| GM12890 | H1ap1 | 0.7 | 73A; 146T; 195T; 247G; 299_314del; 769G; 825T; 1018G; 2706A; 2758G; 2885T; 3010A; 3144G; 3594C; 4104A; 4312C; 7028C; 7146A; 7256C; 7521G; 8468C; 8655C; 8701A; 9540T; 10398A; 10664C; 10688G; 10810T; 10873T; 10915T; 11719G; 11914G; 12705C; 13105A; 13276A; 13506C; 13650C; 14766C; 15497A; 16129G; 16187C; 16223C; 16230A; 16278C; 16311T | No |
| GM12891 | H1e1a | 1 | 73A; 146T; 152T; 247G; 303_315del; 516del; 606G; 769G; 825T; 1018G; 2706A; 2758G; 2885T; 3010A; 3594C; 4104A; 4312C; 4781G; 5460A; 7028C; 7146A; 7256C; 7521G; 8468C; 8512G; 8655C; 8701A; 8975C; 9540T; 10398A; 10664C; 10688G; 10810T; 10873T; 10915T; 11719G; 11914G; 12705C; 13020C; 13105A; 13276A; 13506C; 13650C; 14766C; 14902T; 16129G; 16187C; 16189T; 16223C; 16230A; 16311T | Yes |
| GM12892 | H13a1a1a | 1 | 73A; 146T; 195T; 247G; 303_315del; 769G; 825T; 1018G; 2259T; 2706A; 2758G; 2885T; 3594C; 4104A; 4312C; 4745G; 6266G; 7028C; 7146A; 7256C; 7337A; 7521G; 8468C; 8655C; 8701A; 9540T; 10398A; 10664C; 10688G; 10810T; 10873T; 10915T; 11719G; 11914G; 12705C; 13105A; 13276A; 13326C; 13506C; 13650C; 13680T; 14766C; 14831A; 14872T; 16129G; 16187C; 16189T; 16223C; 16230A; 16278C; 16311T; 16519T | No |
| GM12893 | H13a1a1a | 1 | 73A; 146T; 195T; 247G; 303_315del; 769G; 825T; 846G; 1018G; 2259T; 2706A; 2758G; 2885T; 3594C; 4104A; 4312C; 4745G; 7028C; 7146A; 7256C; 7337A; 7521G; 8468C; 8655C; 8701A; 9540T; 10398A; 10664C; 10688G; 10810T; 10873T; 10915T; 11719G; 11914G; 12705C; 13105A; 13276A; 13326C; 13506C; 13650C; 13680T; 14766C; 14831A; 14872T; 16129G; 16187C; 16189T; 16223C; 16230A; 16278C; 16311T; 16519T | No |
