# Supplementary material for: MitoRS, a method for high throughput, sensitive, and accurate detection of mitochondrial DNA heteroplasmy
Source: BMC Genomics. 2017 Apr 26;18:326. doi: 10.1186/s12864-017-3695-5 (PMC5405551; doi:10.1186/s12864-017-3695-5)
Supplement: Supplementary file 11 — Sanger sequencing validation for the inheritance of high level heteroplasmy SNV. Slide 1. CEPH family 1463 pedigree chart. Slide 2. Level of heteroplasmy calculated from the MitoRS data, and the Sanger sequencing data in both forward and reverse orientation. MitoRS calculated frequencies are highlighted in red (homoplasmy, > 98%) or orange (high frequency heteroplasmy, between 10% and 98%). The corresponding Sanger frequencies are highlighted in green for easier visualization. Slides 3 to 10. Chromatograms highlighting a difference between the two family members. The positions considered are shown with a black arrowhead. Slide 10. Virtual gel visualization (on a LabChip GX - Perkin Elmer) of the PCR amplification products analyzed by Sanger sequencing. (PPTX 513 kb) [file 12864_2017_3695_MOESM11_ESM.pptx]

## Slide 1
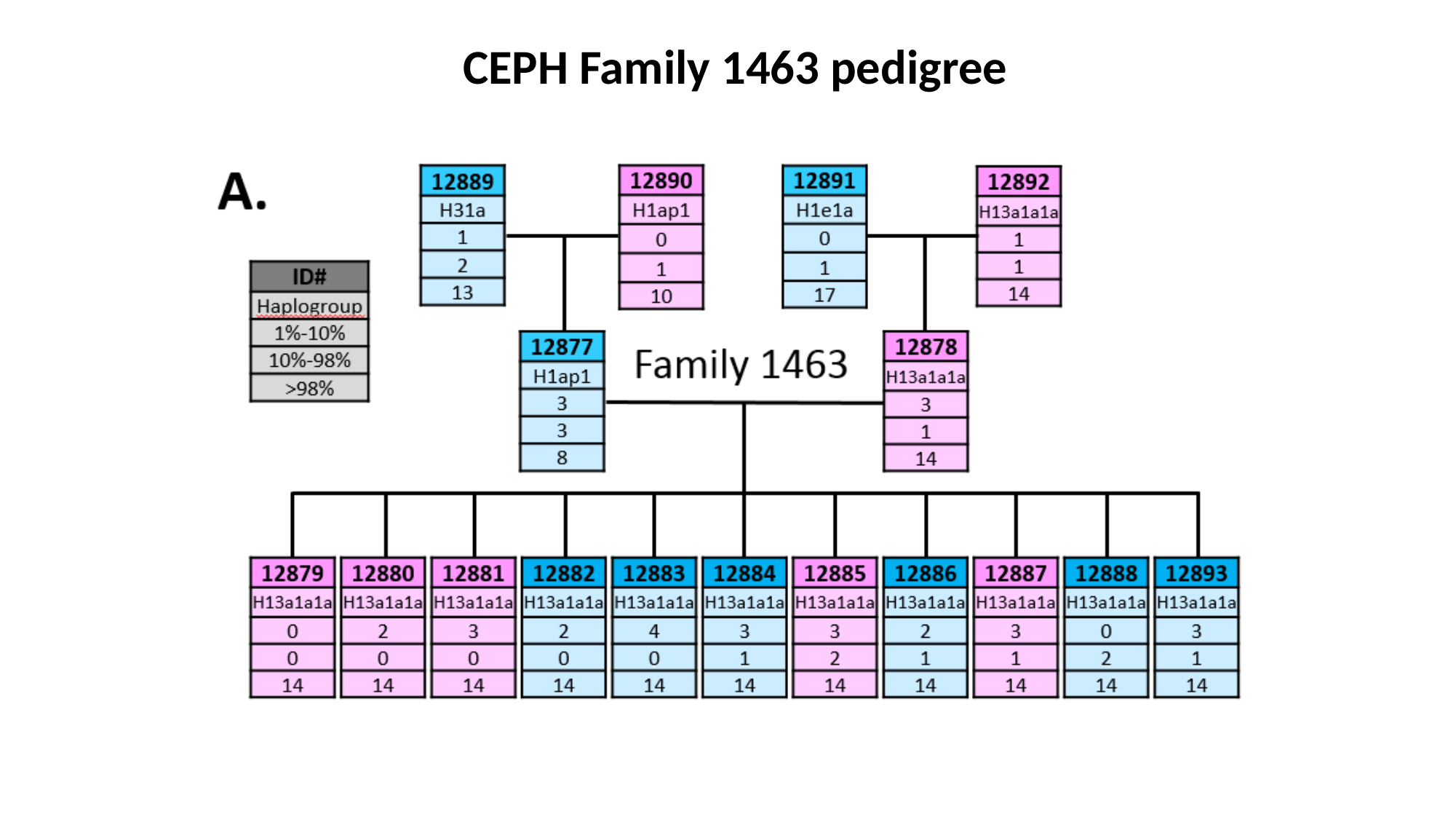

CEPH Family 1463 pedigree

## Slide 2
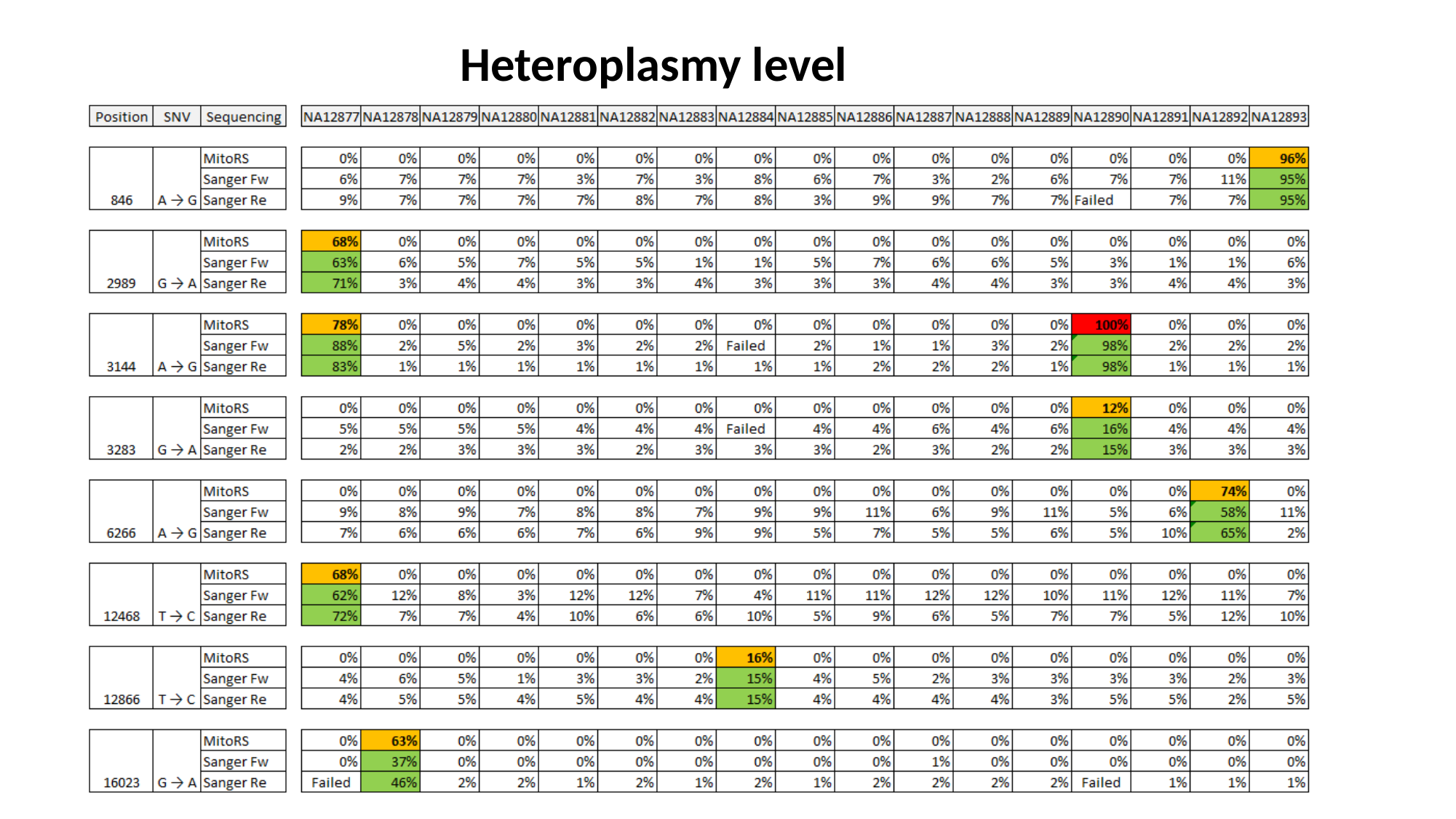

Heteroplasmy level

## Slide 3
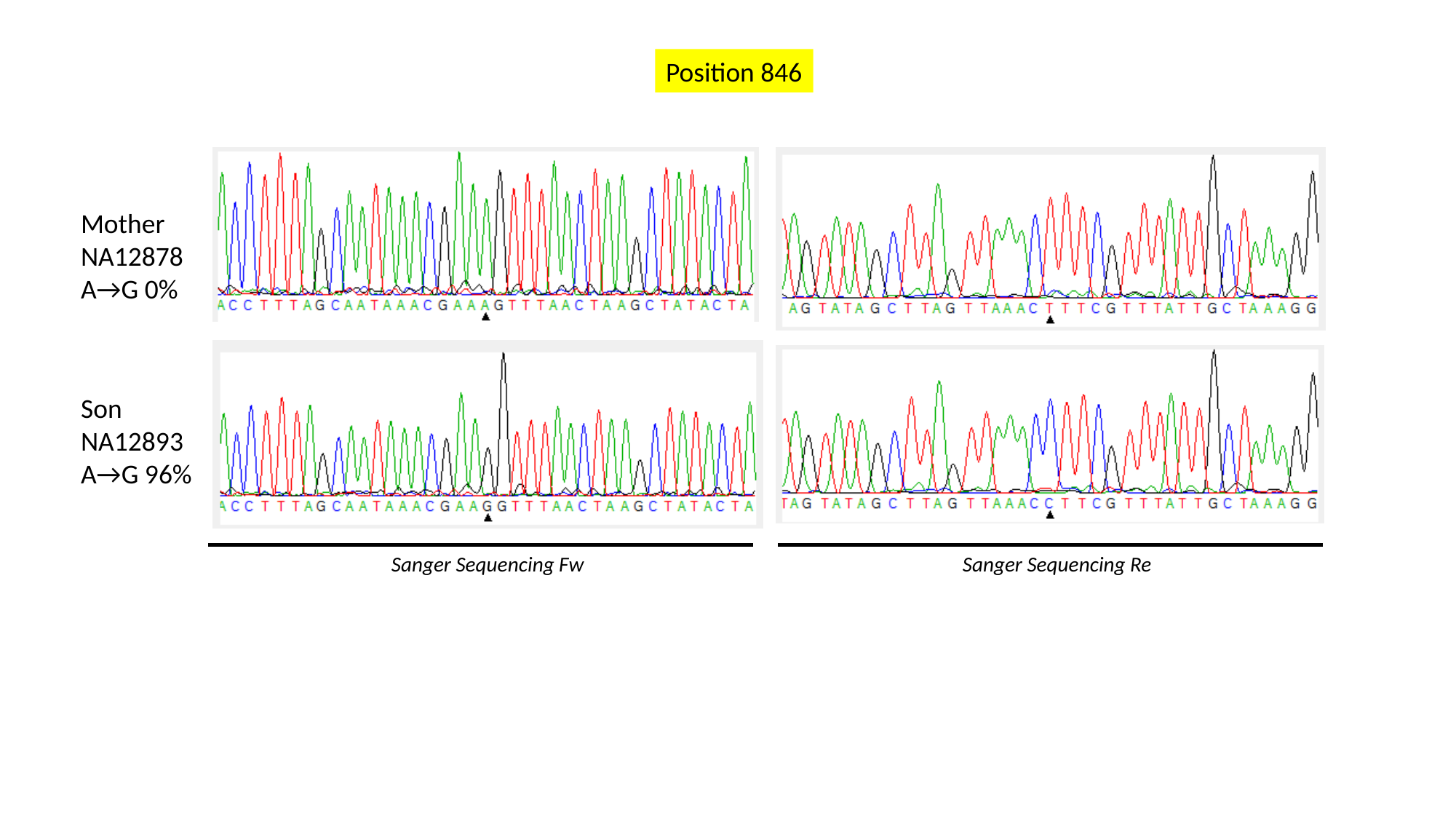

Position 846
Mother
NA12878
A→G 0%
Son
NA12893
A→G 96%
Sanger Sequencing Re
Sanger Sequencing Fw

## Slide 4
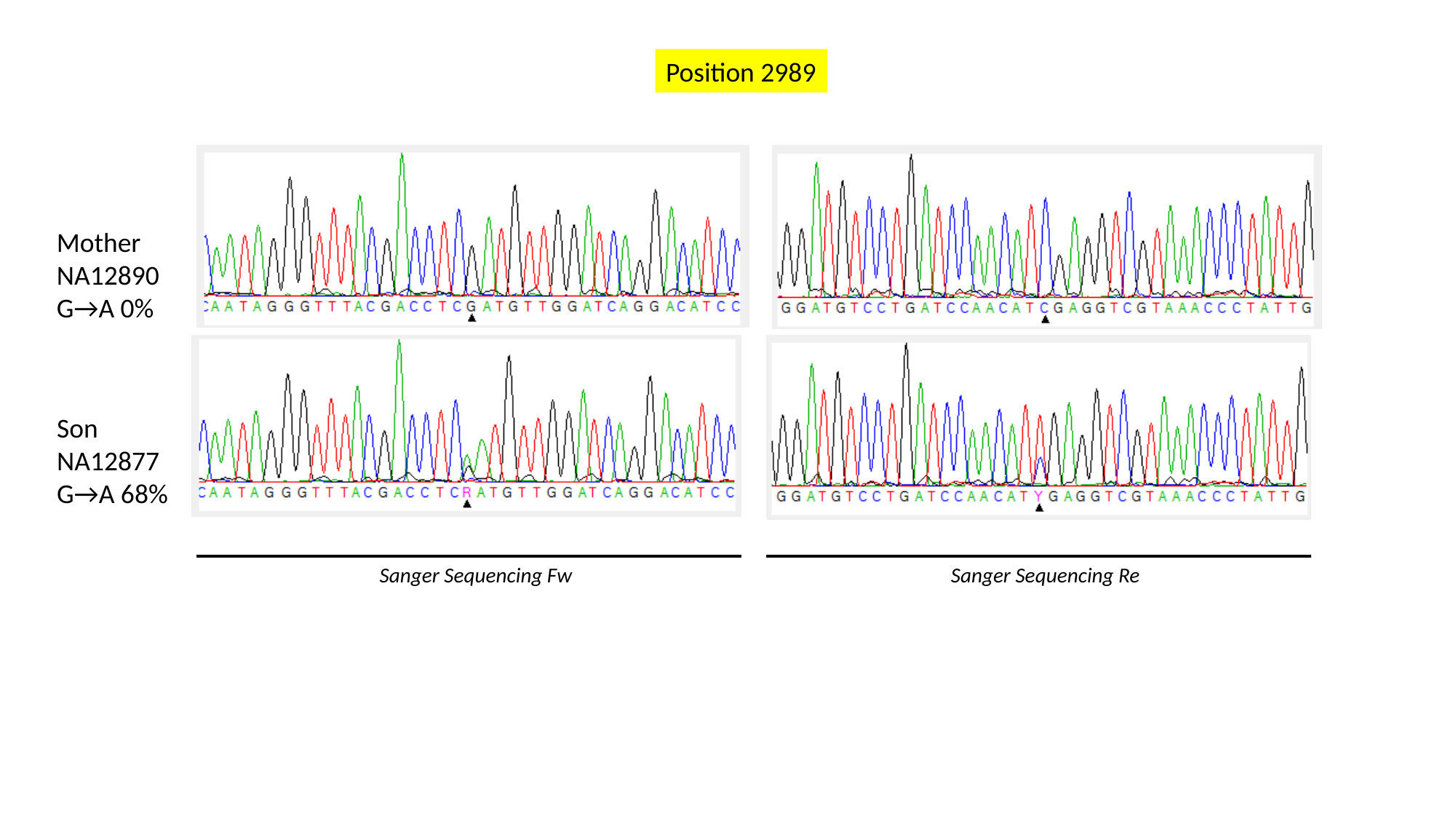

Position 2989
Mother
NA12890
G→A 0%
Son
NA12877
G→A 68%
Sanger Sequencing Re
Sanger Sequencing Fw

## Slide 5
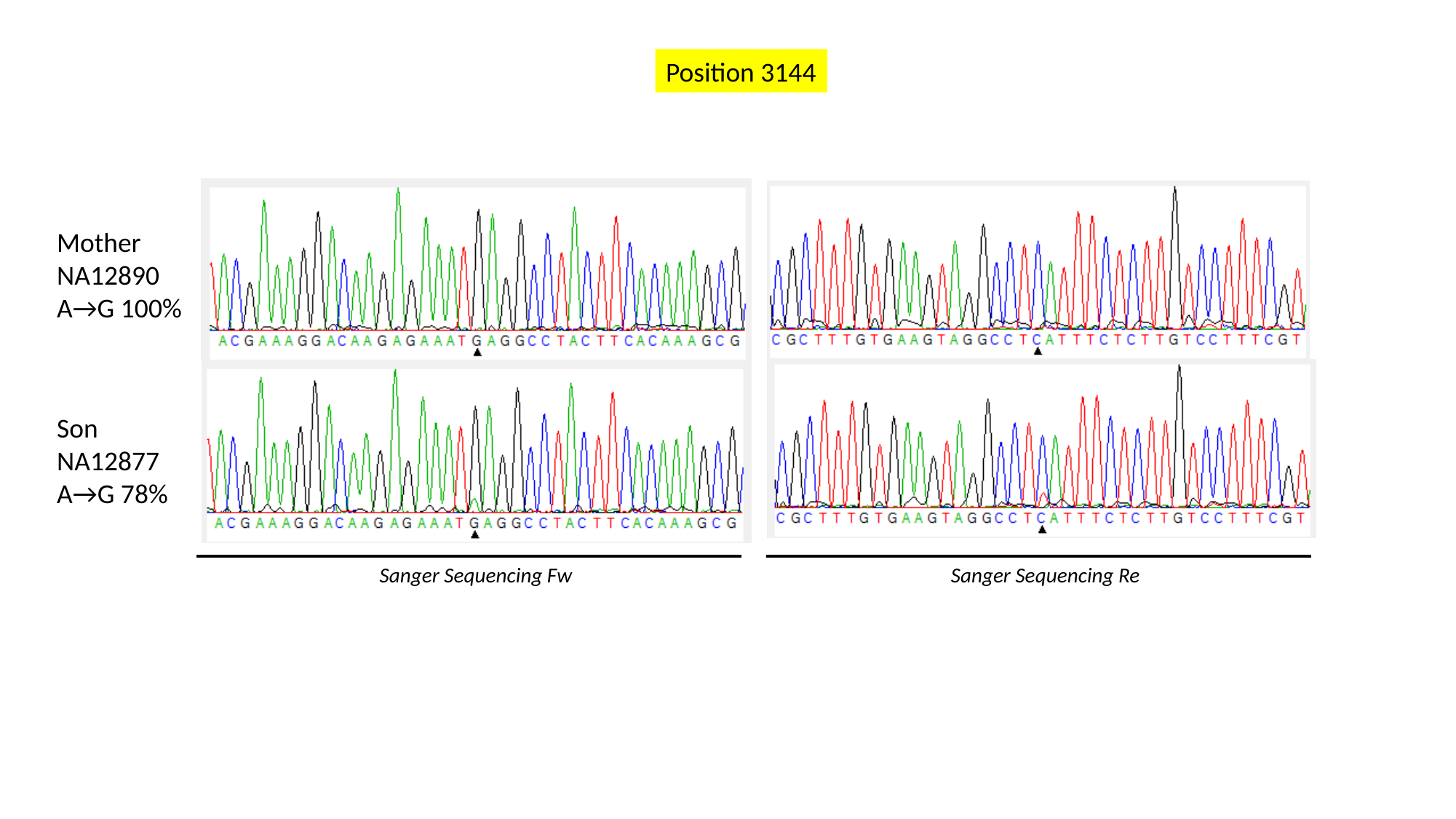

Position 3144
Mother
NA12890
A→G 100%
Son
NA12877
A→G 78%
Sanger Sequencing Re
Sanger Sequencing Fw

## Slide 6
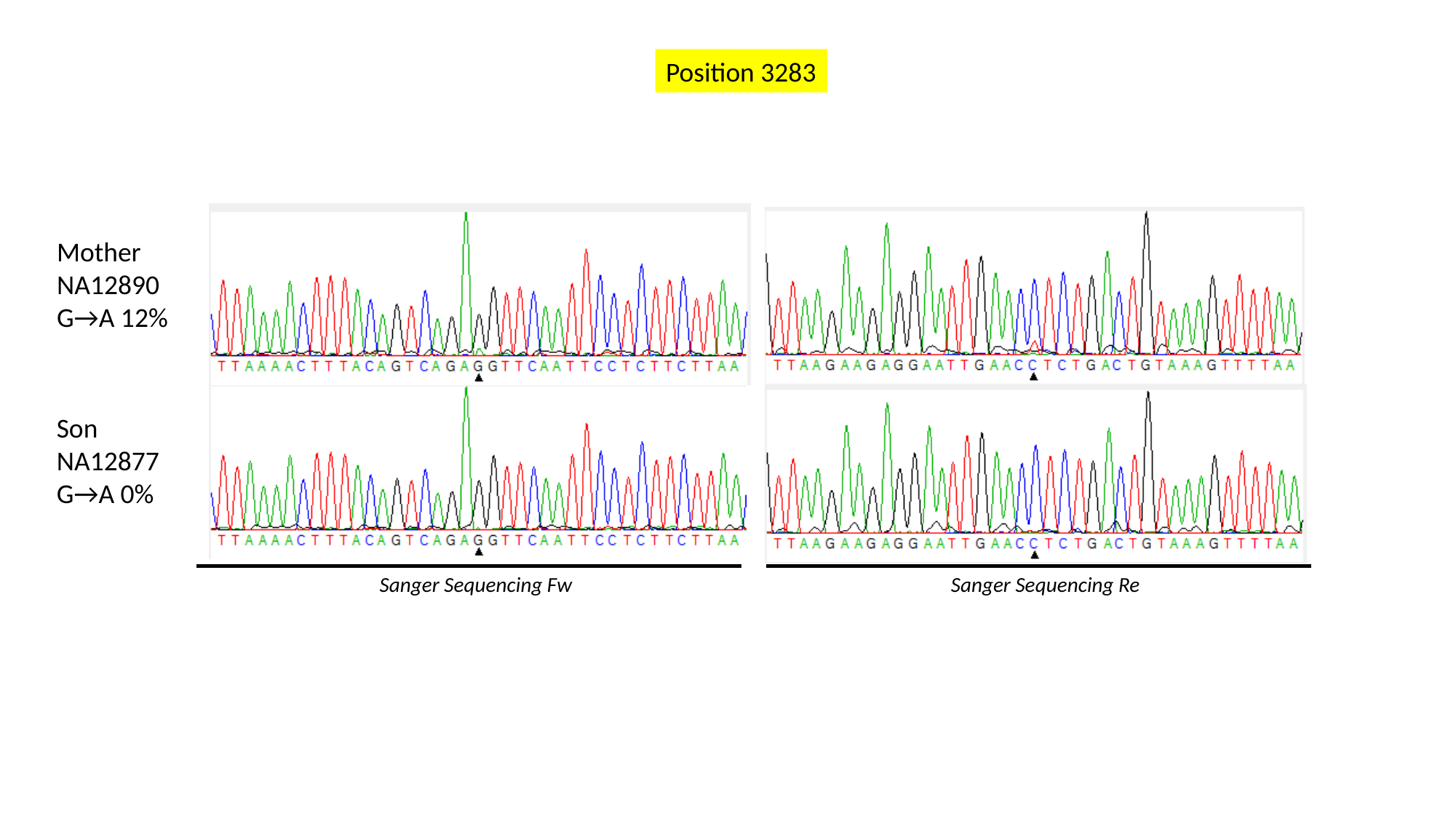

Position 3283
Mother
NA12890
G→A 12%
Son
NA12877
G→A 0%
Sanger Sequencing Re
Sanger Sequencing Fw

## Slide 7
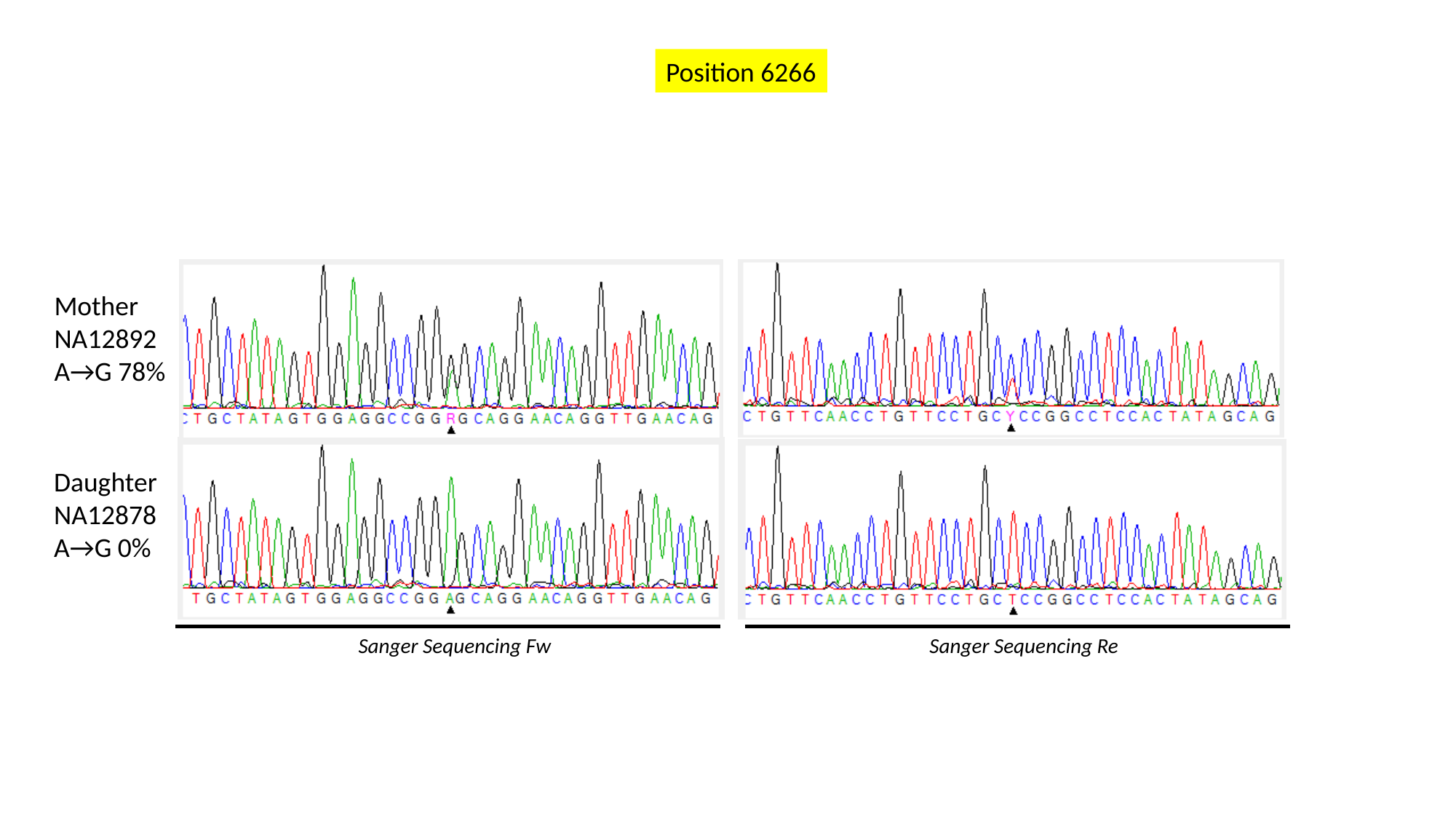

Position 6266
Mother
NA12892
A→G 78%
Daughter
NA12878
A→G 0%
Sanger Sequencing Re
Sanger Sequencing Fw

## Slide 8
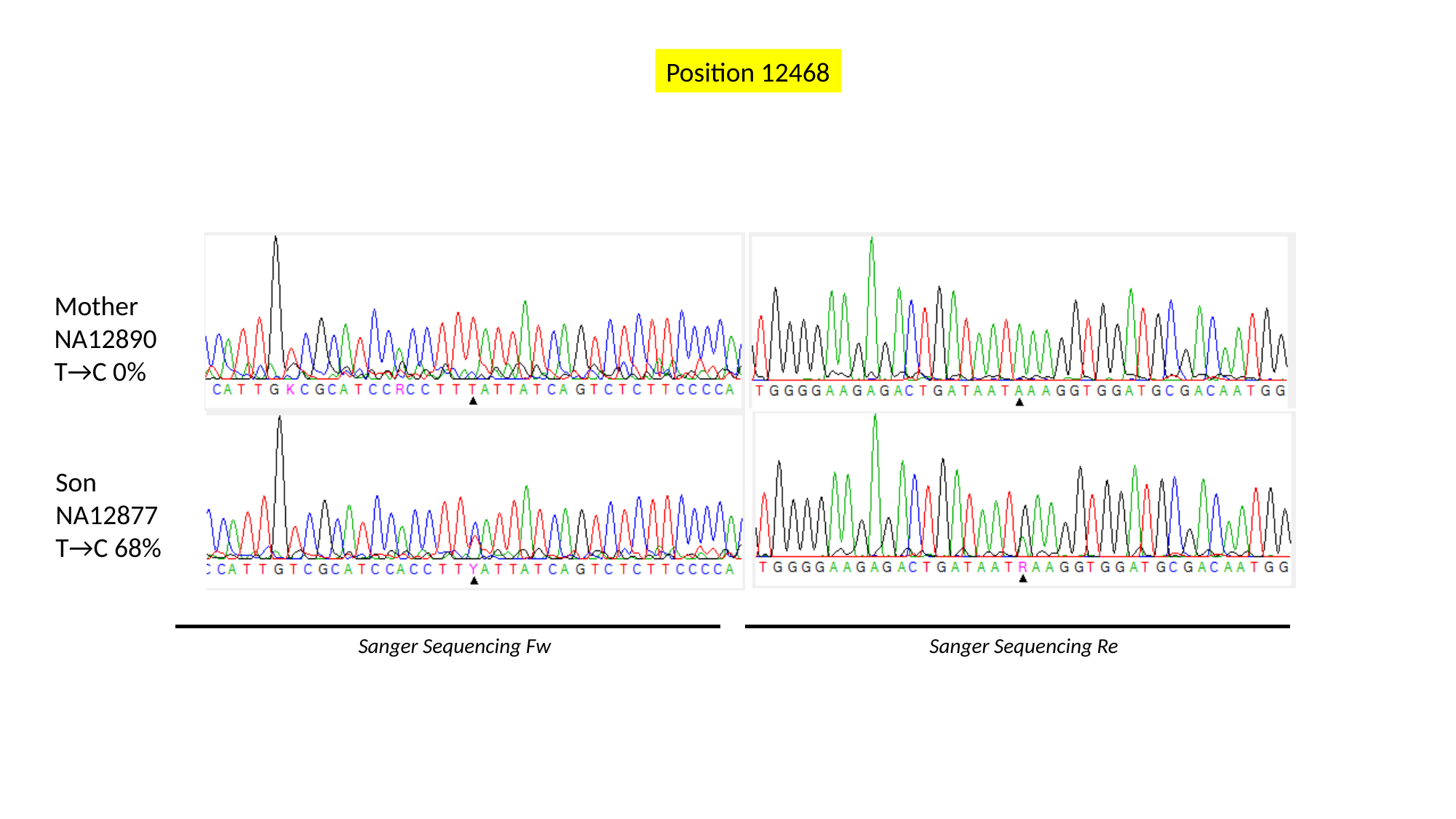

Position 12468
Mother
NA12890
T→C 0%
Son
NA12877
T→C 68%
Sanger Sequencing Re
Sanger Sequencing Fw

## Slide 9
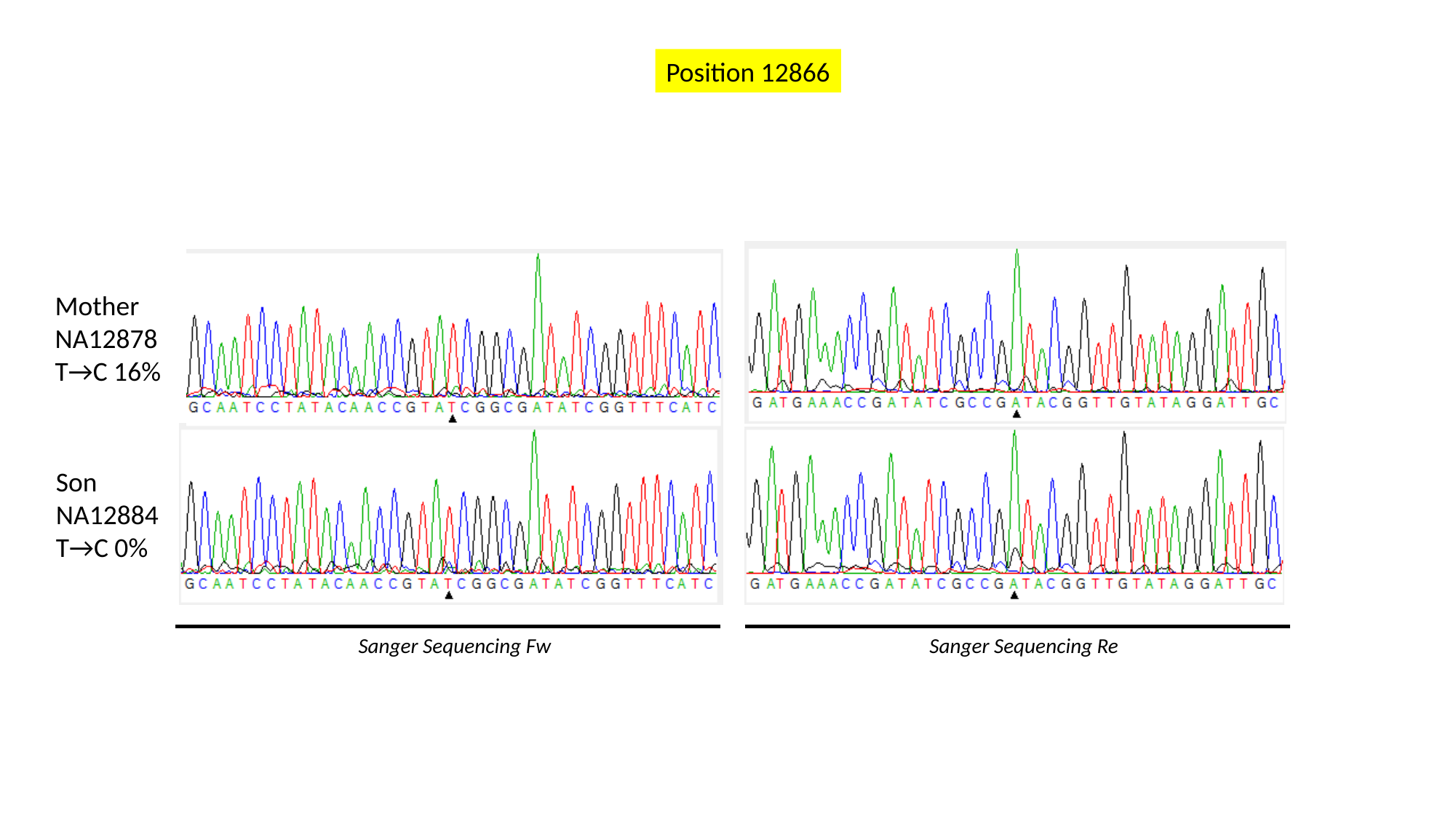

Position 12866
Mother
NA12878
T→C 16%
Son
NA12884
T→C 0%
Sanger Sequencing Re
Sanger Sequencing Fw

## Slide 10
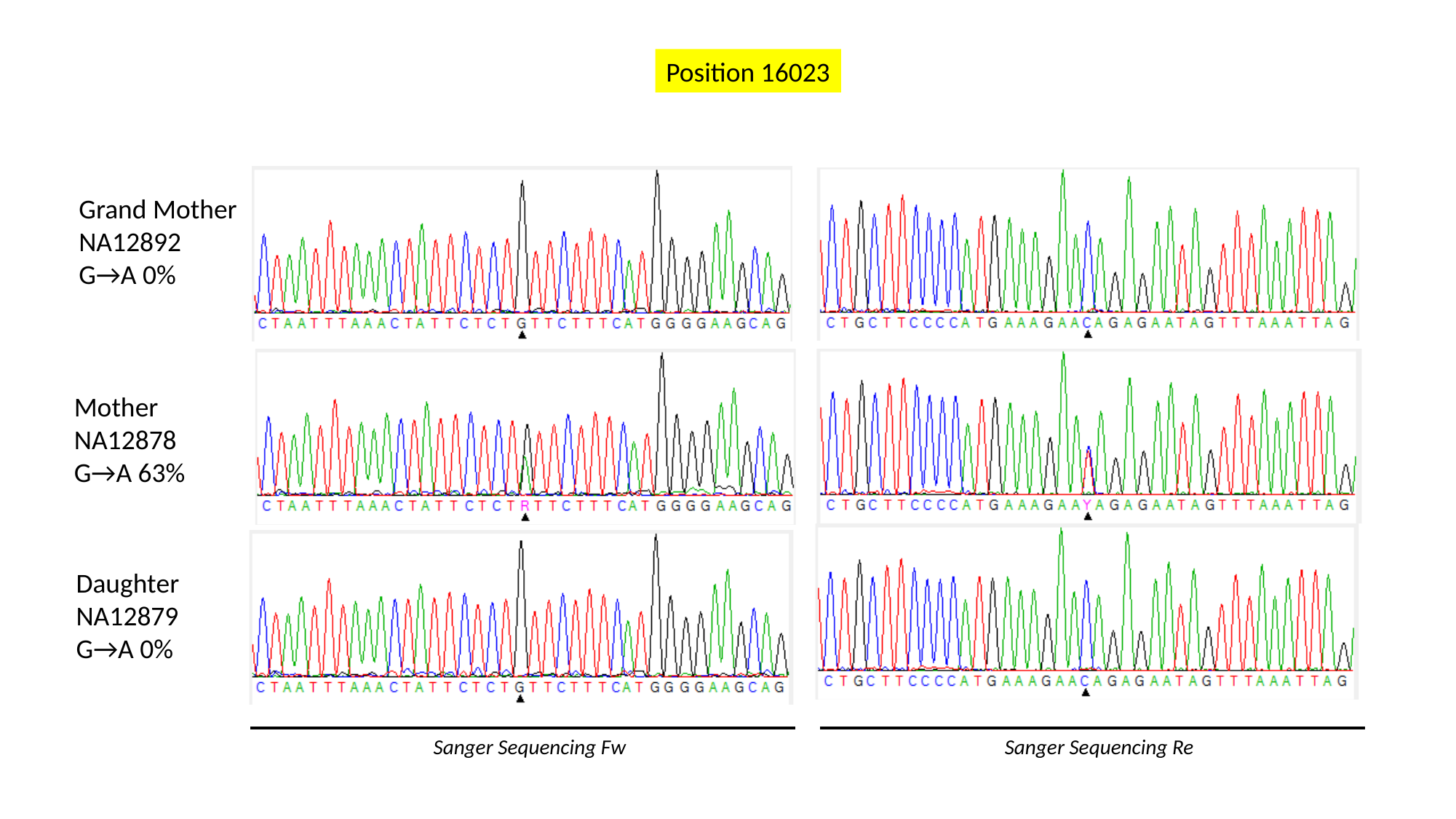

Position 16023
Grand Mother
NA12892
G→A 0%
Mother
NA12878
G→A 63%
Daughter
NA12879
G→A 0%
Sanger Sequencing Re
Sanger Sequencing Fw

## Slide 11
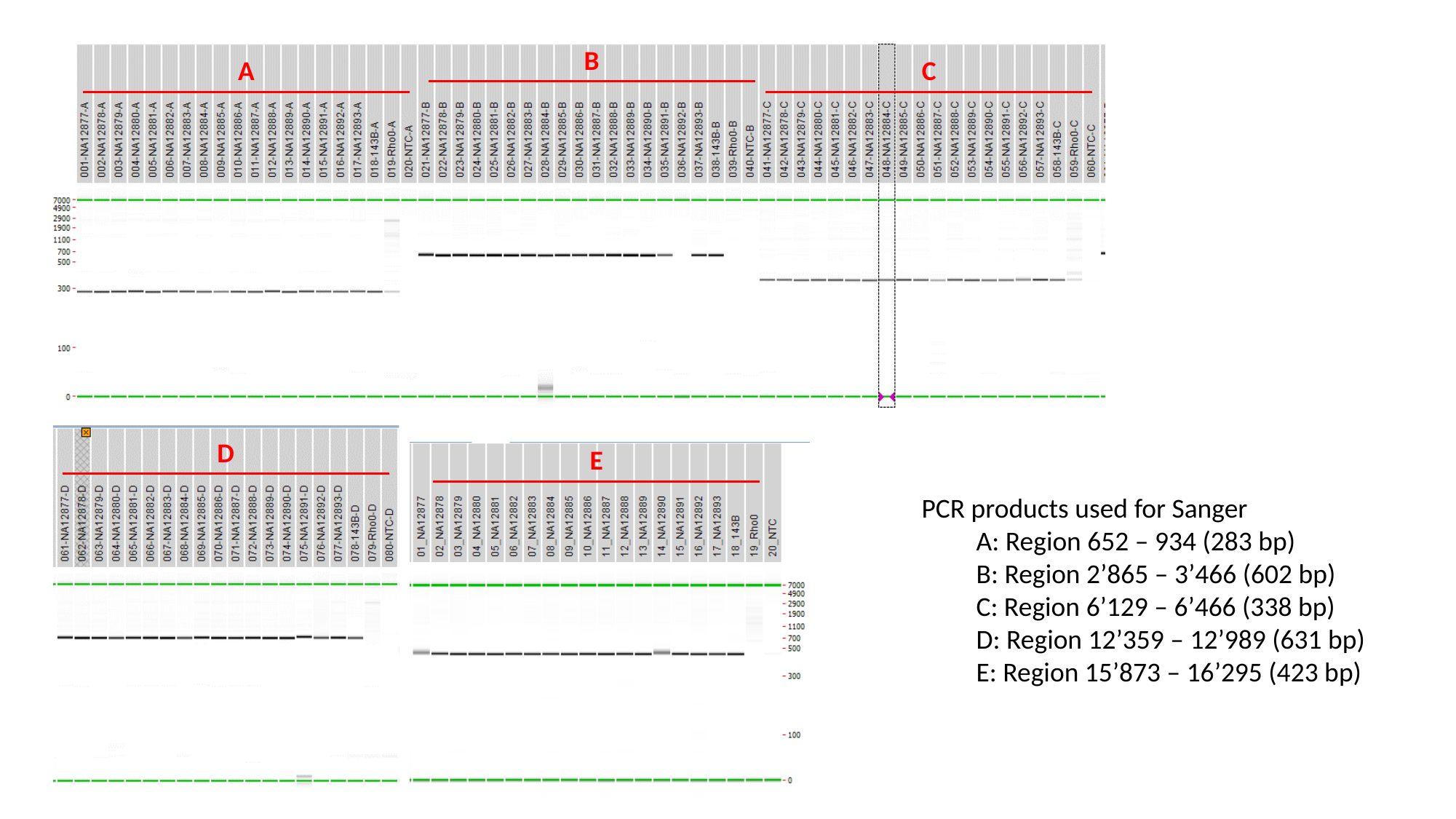

B
A
C
D
E
PCR products used for Sanger
A: Region 652 – 934 (283 bp)
B: Region 2’865 – 3’466 (602 bp)
C: Region 6’129 – 6’466 (338 bp)
D: Region 12’359 – 12’989 (631 bp)
E: Region 15’873 – 16’295 (423 bp)
