# Supplementary material for: MitoRS, a method for high throughput, sensitive, and accurate detection of mitochondrial DNA heteroplasmy
Source: BMC Genomics. 2017 Apr 26;18:326. doi: 10.1186/s12864-017-3695-5 (PMC5405551; doi:10.1186/s12864-017-3695-5)
Supplement: Supplementary file 12 — Primer sequences used for qPCR. (DOCX 37 kb) [file 12864_2017_3695_MOESM12_ESM.docx]

## Table S3

**PCR primer sequences**

| **Primer Name** | **Sequence** |
| --- | --- |
| mm-mtDNA-16S-Fw | CGTCTATGTGGCAAAATAGTGAGAA |
| mm-mtDNA-16S-Re | CCAGCTATCACCAAGCTCGTT |
| mm-mtDNA-16S-Pr | FAM-TAGAGGTGAAAAGCC-MGB |
| mm-mtDNA-ND4-Fw | CACATGGCCTCACATCATCAC |
| mm-mtDNA-ND4-Re | GTGGATCCGTTCGTAGTTGGA |
| mm-mtDNA-ND4-Pr | FAM-CCTATTCTGCCTAGCAAA-MGB |
| mm-PMP22-Fw | TTCGTCAGTCCCACAGTTTTCTC |
| mm-PMP22-Re | ACTCGCTAGTCCCAAGGGTCTA |
| mm-PMP22-Pr | FAM-CGGTCGGAGCATCAGGACGAGC-TAMRA |
| mm-Titin-Fw | AAAACGAGCAGTGACGTGAGC |
| mm-Titin-Re | TTCAGTCATGCTGCTAGCGC |
| mm-Titin-Pr | FAM-TGCACGGAAGCGTCTCGTCTCAGTC-TAMRA |
| hs-mtDNA-16S-Fw | TCGGAGCAGAACCCAACCT |
| hs-mtDNA-16S-Re | ATAGTAGTTCGCTTTGACTGGTGAAG |
| hs-mtDNA-16S-Pr | FAM-CGAGCAGTACATGCTAA-MGB |
| hs-mtDNA-ND4-Fw | CCCTCATTCACACGAGAAAACA |
| hs-mtDNA-ND4-Re | TTGAGGGATAGGAGGAGAATGG |
| hs-mtDNA-ND4-Pr | FAM-CCTCATGTTCATACACCTAT-MGB |
| hs-Alb-Fw | GCTGTCATCTCTTGTGGGCTGT |
| hs-Alb-Re | ACTCATGGGAGCTGCTGGTTC |
| hs-Alb-Pr | FAM-CCTGTCATGCCCACACAAATCTCTCC-TAMRA |
| hs-TFRC-G-Fw | TGAACATGCCACATGCTTTCAT |
| hs-TFRC-G-Re | GCGTAGCTAAGTGAAAAGGTCATAGC |
| hs-TFRC-G-Pr | FAM-CGTGTGCGTAACACCCGAACCAGG-TAMRA |
| Sanger-A-Fw | GGTTTGGTCCTAGCCTTTC |
| Sanger-A-Re | CCGGCTTCTATTGACTTGG |
| Sanger-B-Fw | CACCAGTCAAAGCGAACTAC |
| Sanger-B-Re | TTTATGGCGTCAGCGAAG |
| Sanger-C-Fw | GAGGCTTTGGCAACTGAC |
| Sanger-C-Re | GACGGATCAGACGAAGAG |
| Sanger-D-Fw | CACCCTAACCCTGACTTC |
| Sanger-D-Re | TAGGAGGAGGCCTAGTAGTG |
| Sanger-E-Fw | TACTCAAATGGGCCTGTC |
| Sanger-E-Re | GGTGGGTAGGTTTGTTGGTATC |
