# Supplementary material for: MitoRS, a method for high throughput, sensitive, and accurate detection of mitochondrial DNA heteroplasmy
Source: BMC Genomics. 2017 Apr 26;18:326. doi: 10.1186/s12864-017-3695-5 (PMC5405551; doi:10.1186/s12864-017-3695-5)
Supplement: Supplementary file 14 — Structure of the output csv table. Example of an output file generate by the analysis pipeline. The table is populated for all positions of the reference genome. Chrom: Reference genome used, Position: Position in the reference genome, Covmp: Absolute depth of coverage, PercentCov: relative depth of coverage expressed as a percentage of the average coverage obtained for the sample, FilterCov: Flagging for insufficient relative coverage, Ref: Nucleotide from the reference genome, Var: Alternative nucleotide identified by VarScan, Cons: Consensus nucleotide kept by VarScan, FastA: Nucleotide kept in the exported fastA file, QDepth: Absolute depth of coverage, Reads1: Reference nucleotide coverage by mpileup, Reads2: Alternative nucleotide coverage by mpileup, Freq: Variant frequency, P-value: VarScan p-value, StrandFilter: VarScan strand filter, R1+: Reference nucleotide coverage from the positive strand, R1-: Reference nucleotide coverage from the negative strand, R2+: Alternative nucleotide coverage from the positive strand, R2-: Alternative nucleotide coverage from the negative strand. When several samples are analyzed together, each sample data are populated in consecutive columns of a single table. Full output tables from data presented in this manuscript can be found in the Additional files 7, 8, 9 and 13. (DOCX 46 kb) [file 12864_2017_3695_MOESM14_ESM.docx]

## Table S1

**Structure of the output csv table.**

| Chrom | Position | Covmp | PercentCov | FilterCov | Ref | Var | Cons | Fasta | Qdepth | Reads1 | Reads2 | Freq | P-value | Strand  Filter | R1+ | R1- | R2+ | R2- |
| --- | --- | --- | --- | --- | --- | --- | --- | --- | --- | --- | --- | --- | --- | --- | --- | --- | --- | --- |
| rCRS | 2986 | 1914 | 115% | GOOD | C | . | C | C | 1914 | 1914 | 0 | 0.0% | 1.0E+00 | Pass | 1192 | 722 | 0 | 0 |
| rCRS | 2987 | 1813 | 109% | GOOD | T | . | T | T | 1813 | 1813 | 0 | 0.0% | 1.0E+00 | Pass | 1186 | 627 | 0 | 0 |
| rCRS | 2988 | 1969 | 119% | GOOD | C | . | C | C | 1969 | 1969 | 0 | 0.0% | 1.0E+00 | Pass | 1242 | 727 | 0 | 0 |
| rCRS | 2989 | 2037 | 123% | GOOD | G | A | A | A | 2037 | 661 | 1376 | 67.6% | 0.0E+00 | Pass | 408 | 253 | 834 | 542 |
| rCRS | 2990 | 1940 | 117% | GOOD | A | . | A | A | 1940 | 1940 | 0 | 0.0% | 1.0E+00 | Pass | 1185 | 755 | 0 | 0 |
| rCRS | 2991 | 1954 | 118% | GOOD | T | . | T | T | 1954 | 1953 | 1 | 0.1% | 7.5E-01 | Pass | 1213 | 740 | 1 | 0 |
| rCRS | 2992 | 1939 | 117% | GOOD | G | . | G | G | 1939 | 1939 | 0 | 0.0% | 1.0E+00 | Pass | 1206 | 733 | 0 | 0 |
| rCRS | 2993 | 1821 | 110% | GOOD | T | . | T | T | 1821 | 1820 | 1 | 0.1% | 7.5E-01 | Pass | 1045 | 775 | 1 | 0 |
| rCRS | 2994 | 2005 | 121% | GOOD | T | . | T | T | 2005 | 2004 | 1 | 0.1% | 8.8E-01 | Pass | 1216 | 788 | 1 | 0 |
| rCRS | 2995 | 2036 | 123% | GOOD | G | . | G | G | 2036 | 2036 | 0 | 0.0% | 1.0E+00 | Pass | 1196 | 840 | 0 | 0 |
| rCRS | 2996 | 1943 | 117% | GOOD | G | . | G | G | 1943 | 1943 | 0 | 0.0% | 1.0E+00 | Pass | 1152 | 791 | 0 | 0 |
| rCRS | 2997 | 1901 | 114% | GOOD | A | . | A | A | 1901 | 1901 | 0 | 0.0% | 1.0E+00 | Pass | 1149 | 752 | 0 | 0 |
| rCRS | 2998 | 1891 | 114% | GOOD | T | . | T | T | 1891 | 1891 | 0 | 0.0% | 1.0E+00 | Pass | 1138 | 753 | 0 | 0 |
| rCRS | 2999 | 1969 | 119% | GOOD | C | . | C | C | 1969 | 1967 | 2 | 0.1% | 5.0E-01 | Pass | 1170 | 797 | 0 | 2 |
| rCRS | 3000 | 1953 | 118% | GOOD | A | . | A | A | 1953 | 1953 | 0 | 0.0% | 1.0E+00 | Pass | 1163 | 790 | 0 | 0 |
| rCRS | 3001 | 1966 | 118% | GOOD | G | . | G | G | 1966 | 1966 | 0 | 0.0% | 1.0E+00 | Pass | 1113 | 853 | 0 | 0 |
| rCRS | 3002 | 1758 | 106% | GOOD | G | . | G | G | 1758 | 1758 | 0 | 0.0% | 1.0E+00 | Pass | 1091 | 667 | 0 | 0 |
| rCRS | 3003 | 1717 | 103% | GOOD | A | . | A | A | 1717 | 1717 | 0 | 0.0% | 1.0E+00 | Pass | 1046 | 671 | 0 | 0 |
| rCRS | 3004 | 1839 | 111% | GOOD | C | . | C | C | 1839 | 1839 | 0 | 0.0% | 1.0E+00 | Pass | 1118 | 721 | 0 | 0 |
| rCRS | 3005 | 1764 | 106% | GOOD | A | . | A | A | 1764 | 1764 | 0 | 0.0% | 1.0E+00 | Pass | 1081 | 683 | 0 | 0 |
| rCRS | 3006 | 1773 | 107% | GOOD | T | . | T | T | 1773 | 1771 | 2 | 0.1% | 5.0E-01 | Pass | 1100 | 671 | 1 | 1 |
| rCRS | 3007 | 1841 | 111% | GOOD | C | . | C | C | 1841 | 1841 | 0 | 0.0% | 1.0E+00 | Pass | 1111 | 730 | 0 | 0 |
| rCRS | 3008 | 1836 | 111% | GOOD | C | . | C | C | 1836 | 1836 | 0 | 0.0% | 1.0E+00 | Pass | 1109 | 727 | 0 | 0 |
| rCRS | 3009 | 1902 | 115% | GOOD | C | . | C | C | 1902 | 1900 | 1 | 0.1% | 7.5E-01 | Pass | 1193 | 707 | 0 | 1 |
| rCRS | 3010 | 1945 | 117% | GOOD | G | A | A | A | 1945 | 0 | 1945 | 100.0% | 0.0E+00 | Pass | 0 | 0 | 1192 | 753 |
| rCRS | 3011 | 1881 | 113% | GOOD | A | . | A | A | 1881 | 1881 | 0 | 0.0% | 1.0E+00 | Pass | 1166 | 715 | 0 | 0 |
| rCRS | 3012 | 1745 | 105% | GOOD | T | . | T | T | 1745 | 1744 | 1 | 0.1% | 7.5E-01 | Pass | 1122 | 622 | 1 | 0 |
| rCRS | 3013 | 1817 | 109% | GOOD | G | . | G | G | 1817 | 1817 | 0 | 0.0% | 1.0E+00 | Pass | 1106 | 711 | 0 | 0 |
| rCRS | 3014 | 1748 | 105% | GOOD | G | . | G | G | 1748 | 1748 | 0 | 0.0% | 1.0E+00 | Pass | 1068 | 680 | 0 | 0 |
